# Herb-drug interactions in oncology: pharmacodynamic/pharmacokinetic mechanisms and risk prediction

**DOI:** 10.1186/s13020-025-01156-4

**Published:** 2025-07-07

**Authors:** Xiaoyan Duan, Xiaoyu Fan, Haiyan Jiang, Jie Li, Xue Shen, Zeao Xu, Ziqi Zhou, Jia Xu, Chongze Chen, Hongtao Jin

**Affiliations:** 1 New Drug Safety Evaluation Center, Institute of Materia Medica, Chinese Academy of Medical Sciences & Peking Union Medical College, Beijing, 100050 China; 2 Faculty of Functional Food and Wine, Shenyang Pharmaceutical University, Shenyang, 110016 Liaoning China; 3 College of Life Science and Biopharmaceuticals, Shenyang Pharmaceutical University, Shenyang, 110016 Liaoning China; 4 Department of Dermatology, Beijing Hospital of Traditional Chinese Medicine, Capital Medical University, Beijing, 100010 China; 5 Department of Pharmacy, Fuzhou Changle People’s District Hospital, Fuzhou, 350299 Fujian China; 6 Beijing Union-Genius Pharmaceutical Technology Development Co., Ltd., Beijing, 100176 China; 7 NMPA Key Laboratory for Safety Research and Evaluation of Innovative Drug, Beijing, 100050 China

**Keywords:** Herb-drug interactions, Herbal medicines, Cancer, Pharmacodynamic, Pharmacokinetic, Risk prediction

## Abstract

The prevalence of herbal medicines has gained widespread, particularly among cancer patients seeking adjunctive therapies. Co-administered with anticancer drugs (ACDs) frequently, herbal medicines result in increasing cases of herb-drug interactions (HDIs), following the serious clinical consequences. While herbal medicines pose negative impacts, such as limiting efficacy and increasing toxicity of ACDs, they also offer potential benefits, including enhancing bioavailability, reducing adverse reactions, and reversing tumor drug resistance. This review is the first to systematically characterize HDI molecular mechanisms at both pharmacodynamic (PD) and pharmacokinetic (PK) levels, elucidating how herbal medicines modulate ACDs efficacy and safety through antagonism/synergy/detoxification target, metabolic enzymes, and transporters. In particular, emerging risk prediction methodologies are proposed to assess the clinical occurrence of potential PD/PK-mediated HDIs. We provide a novel insight for promoting the mechanism study of HDIs, facilitating the safe and effective integration of herbal medicines into cancer treatment.

**Graphical Abstract:**

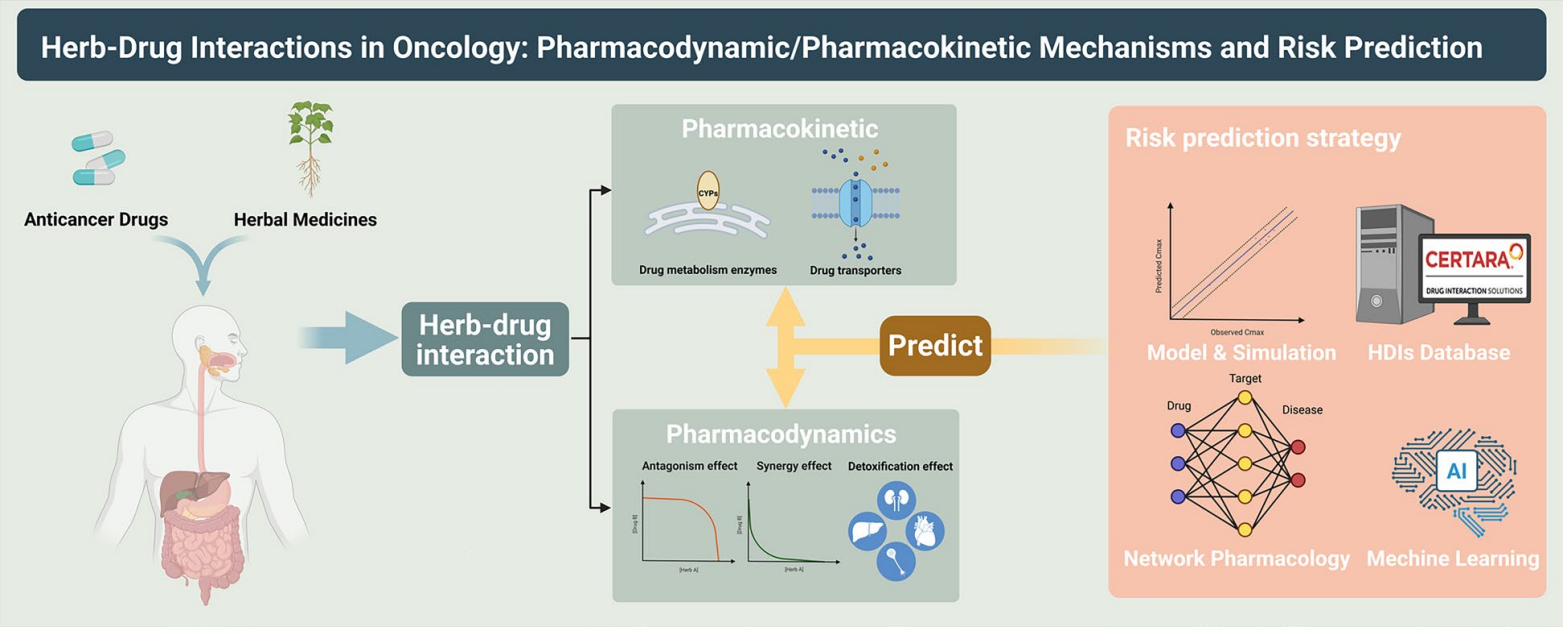

## Introduction

Herbal medicines are becoming increasingly popular as alternative therapies around the world. The usage rate of herbal medicines among the healthy population in the United States was 12% in 1997 and increased to one-third in 2015 [1]. In Africa, 80% of the population has been treated with herbal remedies [2]. In Asian countries such as China, herbal medicines are essential to Traditional Chinese Medicines (TCMs) and are included in standard treatment guidelines for diseases and health insurance systems [3]. According to current World Health Organization statistics, there are a total of 21,000 herbs used for medicinal purposes worldwide [4]. In clinics, these herbal medicines are often co-administered with therapeutic drugs, increasing the potential risk of Herb-drug interactions (HDIs). A comparative study by Prely et al. included 294 patients, of whom 137 (46.6%) took at least one herb, with an average of 1.8 ± 0.8 herbs per patient. A total of 104 HDIs were identified in 68 patients (23.1%), and 36 interactions (34.6%) were identified as high-risk HDIs [5]. The effects of HDIs have a dual role. On the one hand, herbs can cause a decrease in efficacy, selectivity, and even trigger toxicity of therapeutic drugs. For example, *Ginkgo biloba* L. and *Salvia miltiorrhiza* Bunge induce adverse reactions (ADR), such as bleeding when co-administration with warfarin [6, 7]. On the other hand, appropriate application of herbs can increase the bioavailability of therapeutic drugs, improve their efficacy, and reduce ADR. Several studies have shown that *Curcuma longa* L. synergized with doxorubicin (DOX) in suppressing cancer cell proliferation, migration, and invasion, while reducing DOX-induced cardio-nephrotoxicity [8–13]. Researchers and clinicians have long been attempting to appropriately utilize the dual action of HDIs to maximize the clinical benefits of herbal medicines.

Cancer is a life-threatening disease characterized by the uncontrolled growth of abnormal cells, invading vital organs, and severe complications. As anticancer drugs (ACDs), especially chemotherapeutic drugs, have non-specific cell toxicity, cancer patients often experience severe adverse drug reactions [14]. Therefore, they often spontaneously seek alternatives and self-administer herbal therapies. Cancer is the second most common disease associated with herbal use, as reported by Rashrash et al. [1]. More than eight out of ten cancer patients used herbal medicines during chemotherapy, significantly increasing the probability of HDIs [15]. Recent real-world data studies indicated that 45.4% of herbal medicine users were found to be at risk of HDI in oncology treatment [16]. A study in the Middle East found that nearly 400 healthcare workers have prescribed 44 herbal medicines in one year, of which 15 were associated with HDIs with intravenous ACDs [17].

Since most ACDs are cytotoxic with a narrow therapeutic window, small blood concentration changes of drugs induced by herbal medicines are likely to produce profound alterations in efficacy or safety issues. Inappropriate herb-ACDs combinations can lead to severe clinical consequences, such as nausea, vomiting, constipation, diarrhea, pain, hepatotoxicity, nephrotoxicity, etc. [18, 19]. In addition, long-term medication may lead to drug resistance and hampers the efficacy of ACDs. Appropriate herb-ACDs combinations can effectively reverse multidrug resistance (MDR) [20–22]. Therefore, HDIs between herbal medicines and ACDs are more likely to have serious consequences while also offering more potential clinical benefits.

The mechanism of HDIs may involve a combination of pharmacodynamic (PD, antagonism/synergy/detoxification effect on the target) and pharmacokinetic (PK, alteration of the concentration of the ACDs), which in turn leads to alterations in the efficacy or toxicity of ACDs [23]. Thus, it is necessary to elucidate the potential PD/PK mechanisms of herbal medicines-ACDs interactions to avoid adverse effects and improve the safety and efficacy of ACDs. This review provides a first-time characterization and analysis of the mechanism of HDIs in herbal medicines and ACDs at both PD/PK levels. In particular, recent advances in risk prediction methods for HDIs are summarized while presenting the limitations. We aim to provide a novel insight for promoting the mechanism study of HDIs, facilitating the combination of clinical herbs and ACDs, and enriching clinical oncology treatment options.

## Pharmacodynamic-mediated herb-drug interactions

**Fig. 1:**
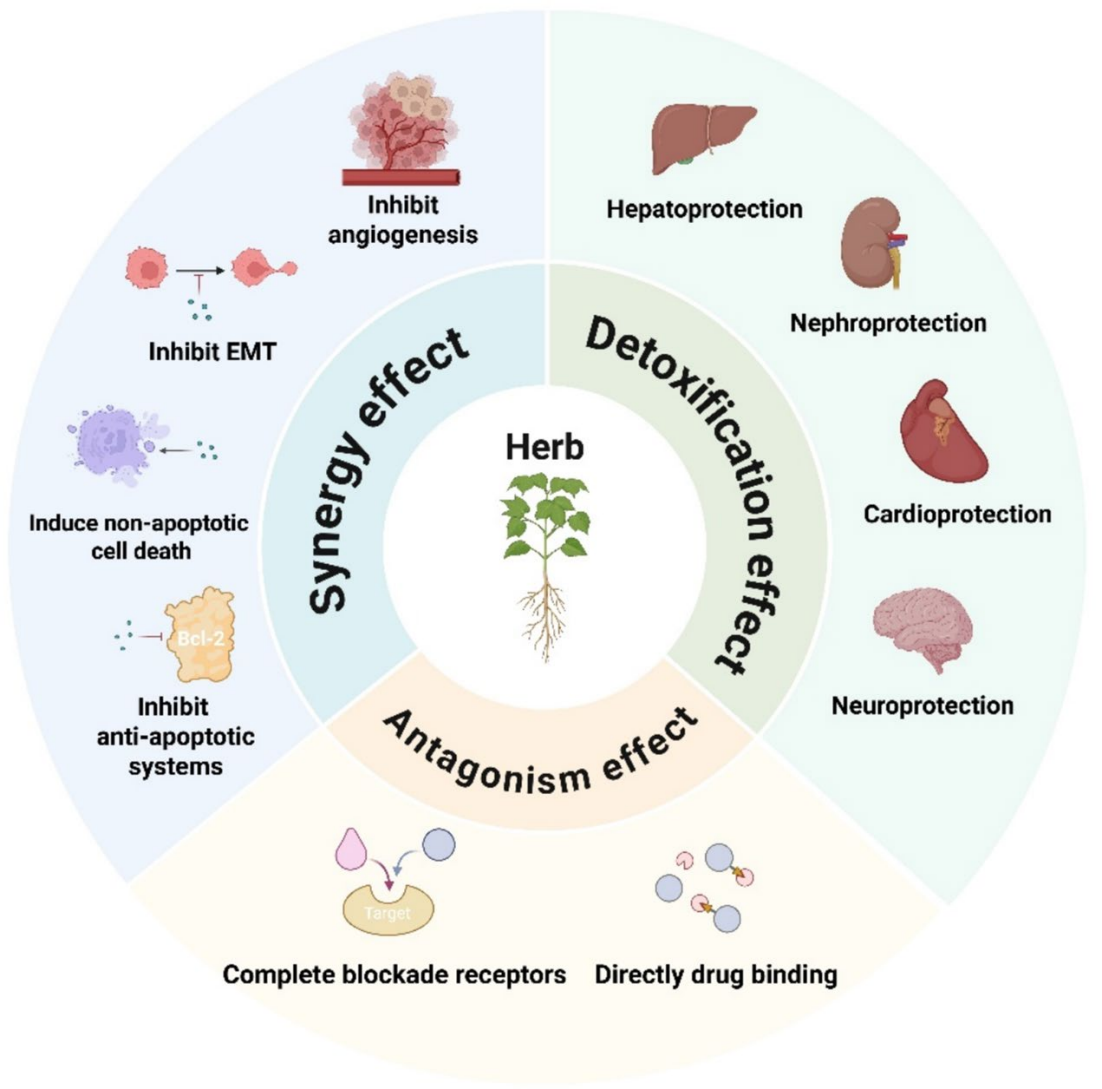
The pharmacodynamic-mediated mechanism of herb-drug interactions

HDIs occur due to competitive or complementary interactions at the same drug target, leading to antagonism or synergy effects between ACDs and herbal medicines. Synergy effects can enhance the therapeutic efficacy of ACDs, but may also lead to adverse toxicity and complicate dosing regimens. In contrast, antagonism effects may result in reduced efficacy and treatment failure. Furthermore, many herbal medicines are reported to reverse the MDR problem associated with the long-term use of ACDs. Some studies also suggest that HDIs can alleviate the ADR of ACDs, exhibiting potential benefits for herbs-ACDs combination. In this section, we explore the PD interactions between herbal medicines and ACDs, focusing on antagonism, synergy and detoxification effects, as shown in Fig. 1.

### Antagonism effect

Antagonism occurs when the overall effect of a drug combination is less than the sum of the pharmacological effects of each individual agent in the combination [24]. Herbal medicines may reduce the potency and toxicity of ACDs by directly binding to the drug or completely blocking specific receptors associated with ACDs. Limit studies have reported antagonism effects of herbal medicines on ACDs, as shown in Table 1. These studies mainly focus on the impact of herbal medicines on cancer cells in vitro. The carbamazepine is a potent histone deacetylase inhibitor of cancer cell growth, and Graviola extract was found to have an antagonism effect when combined with carbamazepine in MCF-7 and PC3 cells [25]. Additionally, in HepG2 cells, it was observed that the combination of curcumin and lovastatin increased the LC_50_ value of lovastatin, suggesting a potential antagonism effect [26]. The combination treatment of *Tamarindus indica* seeds extract with the tamoxifen (TMX) eliminates the cytotoxic effect in MCF-7 cells [27]. Encouse et al. found that green tea components, (−)-epigallocatechin gallate (EGCG), effectively prevent bortezomib (BZM)-induced cancer cells death both in vitro and in vivo [28]. The antagonism effect of EGCG was only evident in boronic acid-based proteasome inhibitors but not in non-boronic acid. Mechanism study indicated that EGCG directly interacts with BZM, blocking its proteasome inhibition function, thereby preventing BZM from triggering endoplasmic reticulum stress or caspase-7 activation.

**Table 1:** Antagonism effect of herbal medicines on ACDs

ACDs	Herbal medicines	Herb type	Subject and dosage	Interaction	Mechanism	References
BZM	EGCG	Active constituents	In vitro; Mice (25–50 mg/kg, p.o.)	Prevent cancer cell death induced by BZM in vitro and in vivo	React with BZM; Block proteasome inhibitory function	[28]
Carbamazepine	Graviola fruit	Herbal extract	In vitro	Decrease cytotoxicity activity of carbamazepine in MCF-7 and PC3 cells	NR	[25]
Lovastatin	Curcumin	Active constituents	In vitro	Decrease cytotoxicity activity of lovastatin in HepG2 cells	NR	[26]
TMX	*Tamarindus indica* L.	Herbal extract	In vitro	Eliminate the cytotoxic effect of TMX in MCF-7 cells	NR	[27]

*BZM* bortezomib, *EGCG* (−)-epigallocatechin gallate, *NR* not reported, *p.o.* oral administration, *TMX* tamoxifen

### Synergy effect and multitarget effect

Tumor progression involves complex molecular networks, making single-target therapies prone to failure due to interpatient heterogeneity and adaptive mechanisms such as target downregulation and MDR. Herbal medicines, characterized by multi-constituents, offer a unique advantage by synergistically acting on the same targets, complementarily unaffected targets, and MDR-related targets. Such multitarget effects underpin the therapeutic potential of herb-drug combinations [29]. Nagaprashantha et al. studied the combination of vicenin-2 (extracted from *Ocimum sanctum*) with docetaxel (DTX) synergistically suppresses prostate cancer progression in vitro and in vivo by inducing apoptosis, inhibiting angiogenesis and epithelial-mesenchymal transition (EMT). The authors reclaimed it is vicenin-2’s multitarget effects that provide a promising combinate therapeutic strategy for prostate cancer [30]. Above all, this section provides a systematic overview of the key mechanisms by which herbal medicines enhance the therapeutic efficacy of ACDs through synergistic interactions, as evidenced by existing studies. Common herbal medicines-mediated synergistic interactions with ACDs are summarized in Table 2.

**Table 2:** Synergy effect of herbal medicines on ACDs

ACDs	Herbal medicines	Herb type	Subject and dosage	Interaction	Mechanism	References
5-FU	Corilagin	Active constituents	In vitro	Increase cytotoxic effects of 5-FU in HCT-8 cells and SW480 cells	Inhibit the expression of GRP 78	[176]
	Curcumin	Active constituents	In vitro; Mice (100 mg/kg, i.p.)	Show synergy effects with 5-FU to MKN1, MKN74 and SNU668 cells and xenograft SNU668 cells tumors	Inhibit JAK/STAT3 pathway	[177]
	Magnolol	Active constituents	In vitro	Inhibit synergistically HeLa cells proliferation and metastasis	Inhibit EMT; Inhibit PI3K/AKT pathways	[44]
	Tangeretin	Active constituents	In vitro; Mice (25 mg/kg, i.p.)	Enhance anticaner effect of 5-FU against HCT116, SW480, SW620, and CT-26 cells and in vivo	Induce autophagy; Inhibit PI3K/AKT pathway	[178]
	*Paris polyphylla* Sm	Herbal extract	In vitro	Synergitize with 5-FU and CDDP in HCT-116	Increase apoptosis	[179]
	*Prunus spinosa* L	Herbal extract	In vitro	Sensitize HCT116 cells to 5-FU	Inhibit PI3/AKT pathways	[180]
	*Scutellaria baicalensis* Georgi	Herbal extract	In vitro; Mice (4 g/kg, p.o.)	Enhance 5-FU-based chemotherapy to CRC cells and in vivo	Inhibit CDK-RB pathway	[181]
	Thai noni juice	Herbal extract	In vitro; Mice (125-250 mg/kg, i.p.)	Synergisticly enhance anti-cancer effect of 5-FU to CCA cells and in vivo	Increase apoptosis; Inhibit anti-apoptotic systems	[182]
	Jiedu Sangen decoction	Herbal prescription	In vitro	Inhibit chemoresistance to 5-FU of HCT-8/5-FU cells	Inhibit tumor glycolysis and PI3K/AKT pathway	[183]
	Fuzheng jiedu Quyu recipe	Herbal prescription	Human (15 g, p.o.)	Prolong mPFS significantly compared with the control group	Inhibit VEGF pathways	[53]
	Kanglaite injection	Herbal prescription	Human (200 mL, i.v.)	Improve DCR, ORR and QOL	Inhibit PI3K/AKT/mTOR pathways	[35]
Afatinib	Ethoxy-erianin phosphate	Active constituents	In vitro	Synergisticly inhibit the proliferation, motion and angiogenesis of HepG2 and HUVECs cells	Inhibit VEGF and EGFR pathways	[184]
Ara-C	Ginsenoside compound K	Active constituents	In vitro	Synergistize cytotoxic effect of Ara-C in THP-1 and U937 cells	Increase apoptosis and DNA damage	[185]
Bevacizumab	Oxymatrine	Active constituents	In vitro	Enhance the anti-tumor effects of Bevacizumab in MDA-MB-231/MDA-MB-468 cells	Inhibt EMT; Deplete stem cells population	[43]
Bortezomib	Solamargine	Active constituents	In vitro; Mice (8 mg/kg, i.p.)	Enhance bortezomib activity in ARP-1 and NCI-H929 cells and xenograft mouse model	Induce autophagy	[186]
Cabazitaxel	Usnic Acid	Active constituents	In vitro	Enhance the efficacy of cabazitaxel to PC3 and DU145 cells	Increase apoptosis	[187]
CDDP	Triptolide	Active constituents	In vitro	Exhibit synergy effect with CDDP in MDA-MB-231 cells	Inhibit insulin-like growth factor 1 signaling	[188]
	β-Asarone	Active constituents	In vitro	Increase sensitization of CDDP in MGC803, SGC7901, and MKN74 cells	Inhibit tumor glycolysis	[61]
	*Centipeda minima* (L.) A. Braun & Asch	Herbal extract	In vitro	Sensitize CDDP- or MMC-induced DNA damage and apoptosis against A549 and H1299 cells	Inhibit FA pathway	[58]
	*Ocimum gratissimum* L.	Herbal extract	In vitro; Mice (40 mg/kg, p.o.)	Sensitize HepG2 cells to the CDDP and in vivo	Inhibit EMT pathway	[189]
	*Paris polyphylla* Sm	Herbal extract	In vitro	Synergitize with 5-FU and CDDP in HCT-116	Increase apoptosis	[179]
	Kanglaite injection	Herbal prescription	Human (200 mL, i.v.)	Improve DCR, ORR, 1-year survival rate, QOL	Inhibit PI3K/AKT/mTOR pathways	[76]
	Fufang Kushen injection	Herbal prescription	Zebrafish (5–50 μg/mL)	Enhance CDDP efficacy in zebrafish model	Inhibit angiogenesis	[54]
			Human (200 mL, i.v.)	Increase the ORR and DCR		[55]
	Shenmai injection	Herbal prescription	In vitro	Enhance CDDP cytotoxicity in CDDP-resistant A549/DDP cells	Inhibit tumor glycolysis and AKT-mTOR-c-Myc pathway	[190]
Cetuximab	Honokiol	Active constituents	In vitro; Mice (100 mg/kg, i.p.)	Synergistic augment cetuximab’s sensitivity to KRAS mutant CRC cells and in vitro models	Inhibit autophagy	[40]
CTX	Parthenolide	Active constituents	In vitro; Mice (40 mg/kg, p.o.)	Synergistize with CTX in LLC cells and improve the survival rate of tumor-bearing mice	Increase apoptosis	[191]
Dichloroacetate	Albiziabioside A	Active constituents	In vitro	Exhibit synergy effect with dichloroacetate in MCF-7 cells	Increase apoptosis; Induce ferroptosis	[192]
DOX	Glycyrrhetinic acid	Active constituents	In vitro	Enhance cytotoxicity in MCF-7 cells, inhibit migration and tube formation	Increase apoptosis; Inhibit VEGF pathway	[51]
	Magnoflorine	Active constituents	In vitro	Sensitize DOX to MCF-7 and MDA-MB-231 cells	Induce autophagy; Inhibit anti-apoptotic systems	[193]
	Oridonin	Active constituents	In vitro	Increase cytotoxic effect in osteosarcoma and MDA-MB-231 cells	Inhibit angiogenesis; Increase apoptosis	[52, 135]
	Parameritannin A-2	Active constituents	In vitro	Increase cytotoxic effects of DOX in HGC27 cells	Increase apoptosis; Inhibit anti-apoptotic systems	[194]
	β-Asarone	Active constituents	In vitro	Synergisticly inhibit proliferation of Raji lymphoma cells	Increase apoptosis; Deplete stem cells population	[195]
	Cuban propolis	Herbal extract	In vitro	Increase cytotoxic effect in LoVo Dox cells	Increase apoptosis	[196]
DTX	Capsaicin	Active constituents	In vitro; Mice (2 mg/kg, i.p.)	Synergisticly inhibit the growth of LNCaP and PC3 cells and reduce the tumor growth of PC3 in vivo	Inhibit PI3K/AKT pathway	[197]
	Polyphyllin VII	Active constituents	In vitro	Enhance the inhibitory effect of DTX in DU-145 cells and DU145/DTX cells	Increase apoptosis; Induce ferroptosis	[198]
	Brazilian green propolis	Herbal extract	In vitro	Enhance the cytotoxicity of DTX alone against MCF-7 cells	Induce necrosis	[199]
Entinostat	Tetrandrine	Active constituents	In vitro; Mice (25 mg/kg, p.o.)	Enhance antitumor effects of entinostat in vitro and in vivo	Increase apoptosis; Inhibit anti-apoptotic systems	[200]
GFT	Nitidumpeptins B	Active constituents	In vitro	Exhibit synergistic antiproliferative activity in acquired GFT-resistant NSCLC	Inhibt YAP expression	[201]
	Yuanhuadine	Active constituents	In vitro	Exhibit a synergistic grwoth-inhibitory activity in GFT-resistant H1299 cells	Inhibit AXL expression	[202]
GMC	Aloe-emodin	Active constituents	In vitro	Increase GMC cytotoxic effects in A549 and NCI-H1299 cells	Induce autophagy	[203]
	*Brucea javanica* (L.) Merr	Herbal extract	Mice (1 g/kg, p.o.)	Reduce tumor growth rate in pancreatic cancer orthotopic xenograft mouse model	Increase apoptosis	[204]
	Aidi injection	Herbal prescription	Human (50–100 mL, i.v.)	Improve significantly ORR, DCR and QOL	Inhibit anti-apoptotic systems; Inhibit EMT	[45]
MMC	*Centipeda minima* (L.) A. Braun & Asch	Herbal extract	In vitro	Sensitize CDDP- or MMC-induced DNA damage and apoptosis against A549 and H1299 cells	Inhibit FA pathway	[58]
MXT	Parthenolide	Active constituents	In vitro; Mice (40 mg/kg, p.o.)	Synergisticly inhibit the growth of LLC cells and reduce tumor growth rate in tumor-bearing mice	Inhibit angiogenesis and NF-kB signaling pathway	[191]
OVA	Gegen Qinlian decoction	Herbal prescription	In vitro; Mice (5 g/kg, p.o.)	Reverse OVA resistance in LoVo/OXAR cells and reduce tumor xenografts	Inhibit YTHDF1-regulated m6A modification of GLS1	[205]
OXA	Genipin	Active constituents	In vitro; Mice (10 mg/kg, i.p.)	Exert synergistic antitumor effects HCT116 and DLD-1 cells and in vivo	Inhibit ROS/ER/BIM pathway	[206]
	Rhein	Active constituents	In vitro	Enhance apoptosis of Panc-1 and MIAPaca-2 cells	Increase apoptosis; Inhibit PI3K/AKT pathway	[207]
Regorafenib	Catalpol	Active constituents	In vitro	Increase cytotoxic effects of regorafenib in HepG2 and HUH-7 cells	Inhibit PI3K/AKT and VEGF pathways	[208]
SOF	EF24	Active constituents	In vitro; Mice (10 mg/kg, i.p.)	Enhance the antitumor effects of SOF in vitro and in vivo	Inhibit angiogenesis	[50]
	Leachianone A	Active constituents	In vitro	Increase cytotoxic effects of SOF to MHCC97H cells	Increase apoptosis; Inhibit anti-apoptotic systems	[209]
	Osthole	Active constituents	In vitro; Mice (100 mg/kg, p.o.)	Synergistic inhibit HCCLM3, HCCLM3-SR, and SK-Hep-1 cells and tumor grough of HCCLM3 cells	Inhibit cholesterol metabolism	[210]
	Tiliroside	Active constituents	In vitro; Mice (20 mg/kg, i.p.)	Enhance the synergistic anti-HCC activity of SOF HepG2, Hep3B, and SMMC-7721 cells and in vivo	Induce ferroptosis	[38]
TMX	*Elephantopus scaber* L.	Herbal extract	In vitro	Increase cytotoxic effects of TMX to MCTS cells	Increase apoptosis	[211]
TMZ	*Crocus sativus* L.	Herbal extract	In vitro	Enhance the antitumor effect of TMZ against C6 glioma rat cell line	Induce autophagy	[212]
TPT	Safranal	Active constituents	In vitro	Enhance the growth inhibitory effects of TPT to HCT116 and A549 cells	Dysregulate the DNA repair machinery	[213]
Trifluorothymidine	Cryptotanshinone	Active constituents	In vitro	Enhance anticancer effect of trifluorothymidine in HGC-27 cells and AGS cells	Inhibit JAK/STAT3 pathway	[214]
VCR	*Bunium persicum*	Herbal extract	In vitro	Synergitize with VCR in MCF-7 and MDA-MB-231 cells	Increase apoptosis; Inhibit anti-apoptotic systems	[34]

*5-FU* 5-fluorouracil, *Ara-C* cytarabine, *CDDP* cisplatin, *CRC* colorectal cancer, *CTX* cyclophosphamide, *DCR* disease control rate, *DOX* doxorubicin, *DTX* docetaxel, *GFT* gefitinib, *GMC* gemcitabine, *i.p.* intraperitoneal injection, *i.v.* intravenous injection, *MMC* mitomycin C, *MTX* methotrexate, *NR* not reported, *ORR* objective response rate, *OXA* oxaliplatin, *p.o.* oral administration, *QOL* quality of life, *SOF* sorafenib, *TMZ* temozolomide, *TPT* topotecan, *VCR* vincristine

#### Inhibit anti-apoptotic systems

Programmed cell death (PCD) is a form of cell death that can be regulated by various biomolecules [31]. PCD includes apoptosis, autophagy, necroptosis, ferroptosis, and pyroptosis [32]. Most ACDs eliminate tumor cells by inducing apoptosis and associated cell death networks. However, tumor cells can evade PCD through dysregulation of apoptotic signals, such as the activation of anti-apoptotic systems, leading to cancer recurrence. In this process, the Bcl-2 protein family plays a critical role. During apoptosis, pro-apoptotic protein Bax translocates to the outer mitochondrial membrane, forming pores that facilitate cytochrome c release into the cytoplasm, activating the caspase cascade and ultimately leading to cell apoptosis. This process is regulated by the transcription factor p53. Tumor cells can upregulate the anti-apoptotic protein Bcl-2 to inhibit Bax activation, evading ACDs-induced apoptosis [33]. Cell proliferation pathways such as PI3K/AKT/mTOR and NF-κB can further enhance anti-apoptotic systems, promoting tumor cell resistance to apoptosis. Recent studies have shown that certain natural products can restore drug-resistant tumor cells’ sensitivity to ACDs by inhibiting anti-apoptotic signals. For example, *Bunium persicum* seed extract synergizes with vincristine (VCR), enhancing VCR-induced cytotoxicity in MCF-7 cells. This effect is likely associated with inhibiting the NF-κB signaling pathway, upregulating the Bax/Bcl-2 ratio and p53 expression, and suppressing anti-apoptotic systems [34]. A meta-analysis showed that Kanglaite injection combined with 5-FU in treating esophageal, gastric, and colorectal cancers was significantly more effective than chemotherapy alone [35]. It can be attributed to the modulation of the PI3K/AKT/mTOR pathway and induction apoptosis by Kanglaite injection [36].

#### Induce non-apoptotic cell death

In addition to inducing apoptosis, herbal medicines can also trigger non-apoptotic cell death. In combination with ACDs, they can kill cancer cells through multiple cell death, overcoming the resistance of tumors induced by a single PCD mechanism. For instance, oridonin enhances the cytotoxicity of 5-FU against renal cancer both in vitro and in vivo by inducing necroptosis [37]. Tiliroside induces ferroptosis, making hepatocellular carcinoma (HCC) cells sensitive to sorafenib (SOF) through the Keap1/Nrf2 pathway [38].

However, it is noteworthy that autophagy appears to exhibit a dual role in the mechanism of herbal synergy. Under stress conditions such as drug exposure and nutrient deprivation, protective autophagy maintains the genomic stability of cancer cells by degrading damaged organelles and proteins, thereby reducing apoptosis induced by ACDs and promoting MDR [39]. Herbal medicines can enhance the cytotoxicity of ACDs by inhibiting protective autophagy and disrupting the survival mechanisms of cancer cells. Honokiol has been shown to inhibit autophagy in KRAS-mutant cells and synergize cetuximab to colorectal cancer (CRC) both in vitro and in vivo [40]. On the other hand, herbal medicines can excessively activate autophagy pathways, leading to excessive self-degradation of cancer cells and triggering autophagic cell death. Zhang et al. reported that oridonin can enhance cellular autophagy by reducing p62 expression and upregulating the conversion of LC3-II. Combined with NVP-BEZ235, it significantly induces apoptosis in neuroblastoma cells and inhibits the growth of neuroblastoma xenografts [41].

#### Inhibit the EMT

EMT refers to the transformation of epithelial cells into mesenchymal cells, which endows cancer cells with the ability to migrate and invade. Drug stimulation triggers EMT, further contributing to tumor progression, metastasis, and drug resistance [42]. Herbal medicines can inhibit EMT, reduce cancer cell migration and invasion, reverse drug resistance, and synergize ACDs. For example, bevacizumab induces EMT in triple-negative breast cancer (TNBC) cells by activating the Wnt/β-catenin pathway, leading to limited efficacy against TNBC. Oxymatrine reverses the EMT phenotype, depletes the bevacizumab-induced TNBC stem cell subpopulation, and enhances the antitumor effect of bevacizumab both in vitro and in vivo [43]. Similarly, magnolol can target the EMT and PI3K/AKT/mTOR pathways to synergistically inhibit cervical cancer cell metastasis when combined with 5-FU [44]. In non-small cell lung cancer (NSCLC) patients, the combination of Aidi injection and gemcitabine significantly improved the objective response rate (ORR) and disease control rate (DCR), possibly be related to inhibiting cell migration and invasion by Aidi injection [45].

#### Inhibit angiogenesis

Oxygen is critical for energy metabolism, driving cellular bioenergetics. The rapid and uncontrolled growth of tumors restricts oxygen availability, making inadequate blood flow, or hypoxia, a common characteristic of nearly all solid tumors [46]. Hypoxic conditions result in a more aggressive phenotype of cancer cells, likely due to hypoxia-induced changes in gene expression and subsequent proteomic alterations [47]. Additionally, there is an intriguing correlation between the hypoxic microenvironment and MDR. Sustained drug treatments suppress tumor angiogenic activity, leading to tumor-associated hypoxia, which promotes the selection of resistant cell clones adapted to oxygen and nutrient deprivation, thereby limiting drug efficacy [48]. A key factor in this process is the activation of HIF-1α/VEGF pathways [49]. Herbal medicines could disrupt hypoxia-angiogenesis crosstalk to achieve synergy effects. For example, hypoxia induced by the anti-angiogenic effects of SOF can lead to SOF -resistance in HCC cells. EF24, a structural analog of curcumin, can promote the degradation of cytoplasmic HIF-1α, thereby synergistically enhancing the antitumor effects of SOF in vitro and in vivo [50]. Additionally, Oridonin and glycyrrhetinic acid have been reported to inhibit angiogenesis, downregulate VEGFR, and increase DOX sensitivity [51, 52]. Fuzheng jiedu Quyu recipe inhibited VEGF and significantly improved progression-free survival combined with 5-FU in the real world [53]. Fufang Kushen injection has been reported to inhibit angiogenesis and significantly improve ORR and DCR in NSCLC patients when combined with platinum-based chemotherapy [54, 55].

#### Other possible synergy mechanism

Chemotherapy typically kills cancer cells by causing DNA damage, such as DNA double-strand breaks or crosslinking. Cancer cells utilize the DNA repair mechanism mediated by the fanconi anemia pathway (FA) to resist the damaging effects of DNA crosslinker [56, 57]. Fan et al. discovered that *Centipeda minima* ethanol extract can inhibit the formation of FANCD2 in the FA pathway, thereby making NSCLC more sensitive to DNA damage and apoptosis induced by cisplatin (CDDP) or mitomycin C (MMC) both in vitro and in vivo [58]. It has been recently shown that cancer stem cells (CSCs) also participate in cancer MDR [59]. CSCs are in the G0 quiescent state of the cell cycle, allowing them to evade the cytotoxic effects of ACDs that target proliferating cells. Additionally, CSCs can reduce the efficacy of ACDs by highly expressing P-gp, inducing anti-apoptotic systems [60]. Drug stimulation regulates the expression of stem cell-related genes and promotes the activation and enrichment of tumor stem cells, ultimately leading to MDR. β-asarone could increase the sensitization of DOX in gastric carcinoma cell lines by abolishing DOX-induced enrichment of the stem-like population and inducing apoptosis [61].

### Detoxification effect

ACDs induce cancer cell death. However, the same mechanisms can also damage normal cells, resulting in side effects and a decline in quality of life. Cancer patients receiving ACDs may experience a variety of ADR, including fatigue, nausea, vomiting, mucositis, alopecia, dry skin, rashes, bowel changes, reduced blood cell counts, and an increased risk of infection [62]. Tissue damage is the most serious side effect of ACDs, leading to various organs toxicity, including kidneys, liver, heart, nerves, etc. [63]. Reducing ACDs-related organ toxicity is of critical importance [64]. Several clinical trials have demonstrated that TCMs can serve as an adjuvant therapy to ACDs and mitigate ADR [35, 45, 55, 65, 66]. Based on ACDs’ special organ toxicity, herbal medicines can provide protective effects for different tissues and organs, as shown in Table 3.

**Table 3:** Detoxification effect of herbal medicines on ACDs

ACDs	Herbal medicine	Herb type	Subject and dosage	Interaction	Mechanism	References
5-FU	*Bletilla striata* polysaccharide	Active constituents	Mice (120 mg/kg, i.p.)	Ameliorate the toxic and side effects of 5-FU in the intestinal tract and bone marrow	Regulate nucleotide synthesis, inflammatory damage, and hormone production	[215]
	Martynoside	Active constituents	In vitro; Mice (20 mg/kg, p.o.)	Protect against 5-FU-induced myelosuppression in vitro and in vivo	NR	[216]
	Xianglian Pill	Herbal prescription	Mice (2.5–0.625 g/kg, p.o.)	Alleviate its gastrointestinal side effects	Regulate the p38 MAPK/NF-κB pathway	[217]
	Kanglaite injection	Herbal prescription	Human (200 mL, i.v.)	Reduce AEs such as vomiting, diarrhea, hematoxicity, hepatotoxicity, neurotoxicity	NR	[35]
	Fuzheng jiedu Quyu recipe	Herbal prescription	Human (15 g, p.o.)	Lighter AEs and reduce the incidence of grade III-IV AEs	NR	[53]
CDDP	Betulin	Active constituents	Rats (8 mg/kg, i.p.)	Reverses CDDP-induced liver injury	Inhibit apoptosis and the NLRP3 inflammasome pathway	[78]
	Chiisanoside	Active constituents	Mice (25–100 mg/kg)	Reverse CDDP-induced ototoxicity	Regulate actin homeostasis; Inhibit ferroptosis	[218]
	Curcumin	Active constituents	Mice (100 mg/kg, i.p.)	Prevent CDDP-induced renal inflammatory injury	Modulate the NF-κB signaling pathway	[70]
	Panduratin A	Active constituents	In vitro; Mice (50 mg/kg)	Protects against CDDP-induced nephrotoxicity	Diminish mitochondrial dysfunction and ROS generation	[71]
	Umbelliferone	Active constituents	Mice (40 mg/kg, i.p.)	Prevent CDDP-induced nephrotoxicity	Regulate Nrf2 pathway	[72]
	Ursolic acid	Active constituents	Mice (80 mg/kg, i.p.)	Attenuate CDDP-induced hearing loss	Inhibit the TRPV1/Ca^2^+/calpain oxidative stress pathway	[219]
	Kanglaite injection	Herbal prescription	Human (200 mL, i.v.)	Reduce severe toxicities by 59%, including hematoxicity, vomiting, neurotoxicity and hepatotoxicity	NR	[76]
	Fufang Kushen injection	Herbal prescription	Human (200 mL, i.v.)	Reduce the frequency of gastrointestinal reaction, hepatoxicity and hematoxiciy,	NR	[55]
	*Salvia officinalis* L.	Herbal extract	Rats (250 mg/kg, p.o.)	Ameliorate CDDP-induced hepatotoxic effects	NR	[77]
CPT-11	Kangai injection	Herbal prescription	Rats (4 mL/kg, i.v.)	Alleviate the severe weight loss induced by CPT-11 in tumor-bearing mice	NR	[220]
CTX	*Pithecellobium dulce* (Roxb.) Benth	Herbal extract	Mice (40 mg/kg, p.o.)	Overcome CTX-induced immunosuppression accompanied with urotoxicity, hepatotoxicity, and nephrotoxicity	NR	[221]
DOX	Glycyrrhetinic acid	Herb active constituents	In vitro; Zebrafish Embryo (40 μM); Mice (40 mg/kg, p.o.)	Protect the heart from DOX-induced cardiotoxicity	Inhibit ferroptosis	[85]
	Hyperoside	Active constituents	Mice (15–30 mg/kg, p.o.)	Prevent DOX-induced cardiotoxicity	Inhibit the NOXs/ROS/NLRP3 inflammasome signaling pathway	[84]
	*Piper nigrum* L.	Herbal extract	Rats (100–200 mg/kg, p.o.)	Ameliorate DOX toxicity of blood chemical and immunological properties in mammary tumor rats	NR	[222]
	Orange peel	Herbal extract	Mice (50 mg/kg, i.p.)	Protect the cellular toxicity of DOX	Inhibit cellular apoptosis	[223]
	*Tripterygium* glycoside	Active constituents	Rats (10 mg/kg, p.o.)	Ameliorate NS induced by DOX in rats	NR	[73]
	Qishen Granule	Herbal prescription	in vitro; Mice (1.67–6.66 g/kg, p.o.)	Protect against DOX-induced cardiotoxicity	Coordinate MDM2-p53-mediated mitophagy and mitochondrial biogenesis	[82]
	Shenmai injection	Herbal prescription	Rats (4.5–9 mL/kg, i.v.)	Alleviate the myocardial injury induced by DOX	Inhibit myocardial autophagy	[81]
GMC	Aidi injection	Herbal prescription	Human (50–100 mL, i.v.)	Reduce the risk of gastrointestinal toxicity, hepatotoxicity, nephrotoxicity	NR	[45]
MTX	*Morinda officinalis* iridoid glycosides	Active constituents	Rats (50–100 mg/kg, p.o.)	Attenuate MTX induced-liver injury	Reverse metabolism disturbance, inhibit the apoptosis and increase the formation of autophagosome	[79]
	Egyptian propolis	Herbal extract	Mice (500 mg/kg, i.p.)	Improve the hepatic and renal biochemical and toxicity parameters of MTX in EAC-bearing mice	NR	[224]
OXA	Curcumin	Active constituents	Mice (10 mg/kg, i.p.)	Alleviate OXA-induced neuropathic pain	Enhance Nrf2-antioxidant responses	[225]
	JianPi-BuShen	Herbal prescription	Human (136 g, p.o.)	Reduce gastrointestinal reaction and neurotoxicity, improve completion rate in chemotherapy	Inhibit pyroptosis	[226]
PTX	Goshajinkigan	Herbal prescription	Human (7.5 mg, p.o.)	Less significantly frequent persistent CIPN 6 months post-chemotherapy	Act on spinal kappa-opioid receptors	[91, 92]

*5-FU* 5-fluorouracil, *AEs* adverse events, *Ara-C* cytarabine, *CDDP* cisplatin, *CIPN* chemotherapy-induced peripheral neurotoxicity, *CPT-11* irinotecan, *CTX* cyclophosphamide, *DOX* doxorubicin, *GFT* gefitinib, *GMC* gemcitabine, *i.p.* intraperitoneal injection, *i.v.* intravenous injection, *MMC* mitomycin C, *MTX* methotrexate, *NR* not reported, *OXA* oxaliplatin, *PTX* paclitaxel, *SOF* sorafenib, *TMZ* temozolomide, *TNBC* triple-negative breast cancer, *TPT* topotecan, *VCR* vincristine

The kidneys are the primary excretory organs in the human body, and most ACDs are eliminated through renal excretion. Consequently, ACDs remain in prolonged contact with various renal regions, leading to nephrotoxicity in different parts. CDDP and methotrexate (MTX) can induce acute kidney injury through direct toxicity to renal tubular epithelial cells, apoptosis activation, oxidative stress, mitochondrial damage, or drug crystallization within renal tubules [67–69]. DOX also causes nephrotic syndrome (NS) by exerting direct toxicity on podocyte cells and inducing oxidative stress and inflammatory responses. Herbal medicines mitigate nephrotoxicity through inhibiting renal cell apoptosis, oxidative stress, and mitochondrial damage. Curcumin prevents CDDP-induced renal inflammatory injury by modulating the NF-κB signaling pathway and reducing the expression of IL-1β, IL-6, IL-8, and TNF-α [70]. Panduratin A, a bioactive compound derived from *Boesenbergia rotunda* (L.) Mansf., protects against CDDP-induced nephrotoxicity by diminishing mitochondrial dysfunction and intracellular ROS generation. [71]. Umbelliferone prevents CDDP-induced nephrotoxicity through regulating Nrf2. [72]. Tripterygium glycoside protects against DOX-induced NS in rats by alleviating podocyte morphological damage, inhibiting caspase-3 activity, and reducing apoptosis [73].

The liver is the primary site for drug metabolism and is significantly exposed to ACDs. Chemotherapeutic agents such as CDDP, MTX and cyclophosphamide (CTX) have been reported to cause hepatotoxicity, which is primarily manifested as elevated transaminase levels, lipid disorders, and cholestasis [74]. Herbal prescriptions Kanglaite injection and Kushen injection have been found in clinical trials to reduce the hepatotoxicity of CDDP significantly and are expected to be used as adjunctive therapies with CDDP [55, 75, 76]. *Salvia officinalis* ethanolic extract effectively ameliorates CDDP-induced hepatotoxicity by reducing liver enzyme activity, alleviating oxidative stress, and improving histopathological changes [77]. By targeting apoptosis and the NLRP3 inflammasome pathway independently of Nek7, Betulin effectively reverses CDDP-induced liver injury [78]. *Morinda officinalis* iridoid glycosides alleviate MTX-induced hepatotoxicity by inhibiting hepatocyte apoptosis, modulating oxidative stress, and reversing lipid metabolism disorders [79]. Pithecellobium dulce extract mitigates CTX-induced hepatotoxicity and nephrotoxicity by improving immunosuppression [77].

Cardiotoxicity is one of the most severe ADR caused by ACDs, contributing to treatment-related mortality. It is commonly observed with anthracycline-based drugs, such as DOX. Certain mechanisms of cardiotoxicity remain unclear but may involve oxidative stress, apoptosis, abnormal expression of related genes, calcium overload, and the generation of toxic metabolites [80]. Some TCMs and natural products have demonstrated protective effects against DOX-induced cardiac dysfunction. Shenmai injection alleviates myocardial injury by inhibiting excessive myocardial autophagy by regulating the miR-30a/Beclin 1 pathway [81]. Qishen Granule protects against DOX-induced cardiotoxicity by coordinating MDM2-p53-mediated mitophagy and mitochondrial biogenesis [82]. Hyperoside protects HL-1 cells from DOX-induced cardiotoxicity by inhibiting the ASK1/p38 signaling pathway and NOXs/ROS/NLRP3 inflammasome signaling pathway [83, 84]. Glycyrrhizic acid protects the heart from DOX-induced cardiotoxicity by activating the Nrf2/HO-1 signaling pathway [85].

Platinum and paclitaxel (PTX) could induce neurological dysfunctions or autonomic nerves, referred to as chemotherapy-induced peripheral neurotoxicity (CIPN) [86]. The incidence of CIPN is predicted to be around 68.1% within the first month following treatment [87]. Oxaliplatin (OXA) causes severe acute and chronic peripheral neuropathies, which may be attributed to altered ion channel activity and significant internal calcium ion depletion [88]. PTX undermines microtubule dynamics, leading to mitochondrial malfunction and the induction of oxidative stress in peripheral neurons [89]. TCMs such as *Astragalus membranaceus* (Fisch.) *Bunge* and *Atractylodes Macrocephala Koidz*. have been widely used in clinical practice to prevent chronic OXA-induced CIPN. This may be attribute to the modulation of the inflammatory response induced by NF-κB on microglial activation [90]. Goshajinkigan (GJG), which consists of 10 types of herbal medicines, showed potential for mitigating CIPN symptoms of PTX in a randomized comparative trial [91]. The mechanism may be GJG’s action on spinal kappa-opioid receptors through dynorphin release, hence diminishing the perception of pain [92].

## Pharmacokinetic-mediated herb-drug interactions

**Fig. 2:**
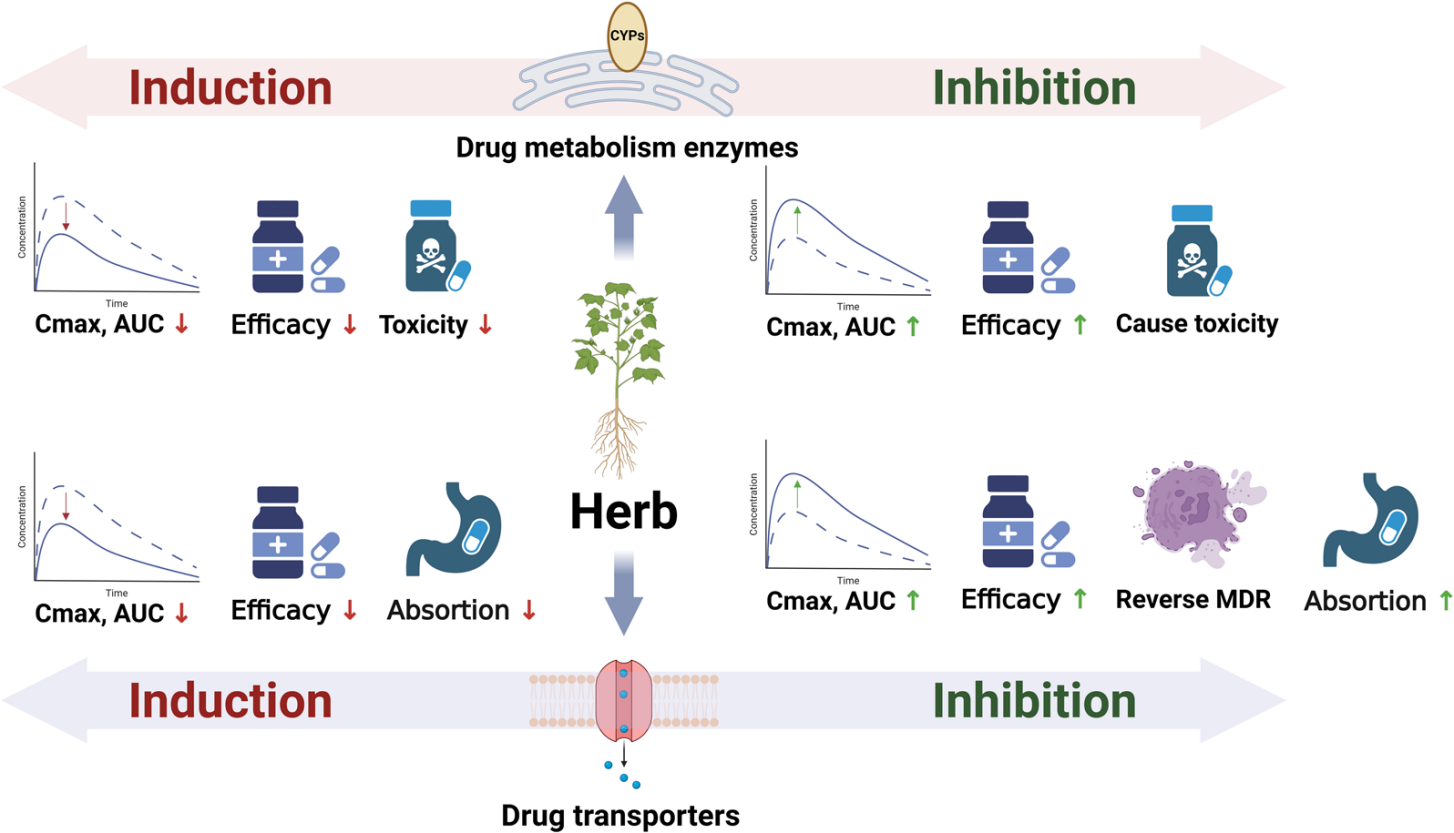
The pharmacokinetic-mediated mechanism of herb-drug interactions

The therapeutic efficacy of ACDs is closely related to their concentration in the tumor or blood circulation. Most ACDs in the clinic, especially cytotoxic drugs, are usually used at the maximum tolerated dose to achieve maximum killing of cancer cells [93]. However, the therapeutic index of ACDs is minimal, and a 20% increase in blood peak concentration (C_max_) or area under curve (AUC) can lead to a substantial increase in toxicity [2]. Specifically, the main cause is the inhibition or induction of drug metabolizing enzymes (DME) and drug transporters (DT). Figure 2 provides a schematic diagram of the PK-mediated HDIs mechanism. A summary of common herbal medicines-mediated pharmacokinetic interactions with ACDs is shown in Table 4.

**Table 4:** Pharmacokinetic interaction of herbal medicines on ACDs

ACDs	Herbal medicines	Herb Type	Subject and dosage	Interaction	Mechanism	References
5-FU	Furanodiene	Active constituents	Zebrafish	Synergistize anti-cancer effect for both MDA-MB-231 cells and BEL-7402 cells xenotransplanted zebrafish	Inhibit P-gp	[227]
	Terpenoids	Active constituents	In vitro; Mice (50 mg/kg, p.o.)	Enhance the chemotherapy sensitivity of HCT-8/Fu to 5-FU; Enhanced 5-FU accumulation in vitro and in vivo	Inhibit P-gp	[228]
Apatinib	Huosu Yangwei oral liquid	Herbal prescription	Rats (10 mL/kg)	Prolong the plasma half-life; Increase AUC	Inhibit CYP1A, CYP2A6, CYP2C8, CYP2C9, CYP2C19, CYP2D6, CYP2E1, CYP3A	[229]
Ara-C	Proanthocyanidin	Active constituents	In vitro	Lower the IC50 of Ara-C and DOX in HL-60/DOX cells	Inhibit MRP1, P-gp, LRP	[230]
CDDP	Astragaloside IV	Active constituents	In vitro; Mice (50 mg/kg, p.o.)	Enhance the antitumor effect of CDDP in HepG2 cells and H22 tumor-bearing mice	Inhibit MRP2	[231]
	Furanodiene	Active constituents	Zebrafish	Synergistize anti-cancer effect for both MDA-MB-231 cells and BEL-7402 cells xenotransplanted zebrafish	Inhibit P-gp	[227]
	Kanglaite injection	Herbal prescription	In vitro	Increase the antitumor effects of CDDP on HepG2 cells	Inhibit P-gp, MRP2, BCRP	[75]
	Wedelolactone	Active constituents	In vitro; Mice (20 mg/kg)	Reduce kidney accumulation; Ameliorate CDDP-induced kidney injury	Inhibit OCT2	[110]
	6-methylflavone	Active constituents	In vitro; Mice (50 mg/kg)	Decrease CDDP-induced cytotoxicity and kidney injury	Inhibit OCT2	[109]
CPT-11	Psoralidin	Active constituents	Mice (500 mg/kg, p.o.)	Increase gastrointestinal toxicity and body weight loss of CPT-11	Inhibit UGT1A1	[103]
	Caffeic acid	Active constituents	Mice (400 mg/L p.o.)	Increase SN-38 glucuronide excretion; Improve the leukopenia, intestinal oxidative stress and inflammation of CPT-11	Induce UGT1A1	[232]
CTX	*Rhizoma paridis saponins*	Active constituents	Rats (200 mg/kg, p.o.)	Reduce CTX anticancer effect and toxicity in hepatocarcinoma model	Inhibit CYP2B6, CYP3A4	[233]
Dasatinib	Sinapic acid	Active constituents	Rats (20 mg/kg, p.o.)	Increase C_max_, AUC, MRT, T_max_, systemic bioavailability; Decrease Vd and CL	Inhibit CYP3A2, P-gp and BCRP	[234]
DOX	*Dioscorea bulbifera* L	Herbal extract	Mice (0.1 mL/10 g, p.o.)	Decrease survival rate, induce elevated levels of toxicity in the heart and kidneys and delay excretion of DOX	Inhibit P-gp	[18]
	Glabratephrin	Active constituents	In vitro; Mice (5 μM, i.v.)	Increase DOX accumulation and cytotoxicity in breast cancer cells; Reduce the growth of Pgp-expressing tumors	Inhibit P-gp	[118]
	Saikosaponin D	Active constituents	In vitro; Mice (10 mg/kg, i.p.)	Enhance DOX anticancer efficacy in vitro and in vivo; Increase DOX accumulation in breast cancer cells	Inhibit P-gp	[235]
	Tetrandrine	Active constituents	In vitro	Increase the cytotoxicity of DOX, VCR and PTX; Increase the intracellular accumulation in KB-C2 cells	Inhibit P-gp	[123]
	Voacamine	Active constituents	In vitro	Increase the cytotoxicity of PTX or DOX in A2780 DX and LoVo DX drug-resistant cells	Inhibit P-gp	[121]
	Algerian propolis	Herbal extract	In vitro	Increase Dox content in MDA-MB-231 cells; Decrease the IC50 of Dox	Inhibit P-gp	[116]
	*Euryops pectinatus* L	Herbal extract	In vitro	Increased the potency of DOX in MCF/Dox, CEM/ADR500 cells and Caco2 cells	Inhibit P-gp	[117]
	Proanthocyanidin	Active constituents	In vitro	Lower the IC50 of Ara-C and DOX in HL-60/DOX cells	Inhibit MRP1, P-gp, LRP	[230]
	*Scabiosa atropurpure* L.	Herbal extract	In vitro	Increase anti-proliferative effects in Caco-2 cells	Inhibit P-gp and MRPs	[120]
	Shenmai injection	Herbal prescription	In vitro; Mice (5 mL/kg, i.v.)	Strengthened the toxicity to MCF-7/DOX cells; Increase intracellular concentrations; Reduce the weight and volume of tumor	Inhibit P-gp	[122]
	Cinnamophilin	Active constituents	In vitro	Enhance cytotoxic effects of DTX, VCR, or PTX in MDR human cervical cancer cell line	Inhibit P-gp	[236]
DTX	Wogonin	Active constituents	Rats (10–40 mg/kg)	Increase C_max_ and AUC of DTX in rats with mammary tumors	Inhibit CYP3A4, P-gp	[237]
	*St John’s wort*	Herbal extract	Human (300 mg)	Decrease AUC; Increase clearance	Inhibit CYP3A4	[238]
	*Marsdenia tenacissima* (Roxb.) Moon	Herbal extract	In vitro	Restore GFT sensitivity in GFT-resistant NSCLC cells	Inhibit CYP3A4, CYP2D6	[239]
GFT	*Trigonella foenum-graecum* L	Herbal prescription	Human	Exacerbate ribociclib-induced hepatotoxicity	Inhibit CYP3A4	[19]
Ribociclib	*St John’s wort*	Herbal extract	Human (300 mg)	Decrease AUC; Reduce C_max_ and half-life	Inhibit CYP3A4	[240]
IM	*Panax ginseng* C. A. Mey	Herbal extract	Human	Cause hepatotoxicity	Inhibit CYP3A4	[96]
	Botryllamide G	Active constituents	Mice (13.4 mg/kg, i.v.)	Increase brain exposure to lapatinib in mice	Inhibit BCRP	[115]
Lapatinib	Luteolin	Active constituents	Rat (20 mg/kg, p.o.)	Decrease MTX-induced cytotoxicity; Increase AUC	Inhibit OATP1B1	[112]
MTX	Isosinensetin	Active constituents	Rat (20 mg/kg, p.o.)	Attenuate MTX-induced nephrotoxicity; Reduce MTX renal concentrations	Inhibit OAT3	[111]
	Dihydromyricetin	Active constituents	In vitro; Mice (100 mg/kg, i.p.)	Restore chemosensitivity (OXA and VCR) in HCT116/OXA and HCT8/VCR cell lines and in vivo	Inhibit MRP2	[241]
OXA	Cinnamophilin	Active constituents	In vitro	Enhance cytotoxic effects of DTX, VCR, or PTX in MDR human cervical cancer cell line	Inhibit P-gp	[236]
PTX	Tetrandrine	Active constituents	In vitro	Increase the cytotoxicity of DOX, VCR and PTX; Increase the intracellular accumulation in KB-C2 cells	Inhibit P-gp	[123]
	Voacamine	Active constituents	In vitro	Increase the cytotoxicity of PTX or DOX in A2780 DX and LoVo DX drug-resistant cells	Inhibit P-gp	[121]
	Xiaoaiping injection	Herbal prescription	in vitro; Mice (20-40 mL/kg)	Enhances anti-tumor effect of PTX in SK-OV-3 cells and xenograft tumor model	Inhibit CYP2C8, CYP3A4, P-gp	[242]
	Xiang-Sha-Liu-Jun-Zi Tang	Herbal prescription	Rats (250 mg/kg)	Increase AUC; Prolong the half-life	Inhibit CYP3A1, CYP3A2, CYP3A4	[243]
	Cinnamophilin	Active constituents	In vitro	Enhance cytotoxic effects of DTX, VCR, or PTX in MDR human cervical cancer cell line	Inhibit P-gp	[236]
VCR	Dihydromyricetin	Active constituents	In vitro; Mice (100 mg/kg, i.p.)	Restore chemosensitivity (OXA and VCR) in HCT116/OXA and HCT8/VCR cell lines and in vivo	Inhibit MRP2	[241]
	Tetrandrine	Active constituents	In vitro	Increase the cytotoxicity of DOX, VCR and PTX; Increase the intracellular accumulation in KB-C2 cells	Inhibit P-gp	[123]

*5-FU* 5-fluorouracil, *Ara-C* cytarabine, *CDDP* cisplatin, *CTP-11* irinotecan, *CTX* cyclophosphamide, *DOX* doxorubicin, *DTX* docetaxel, *GFT* gefitinib, *IM* imatinib, *NSCLC* non-small cell lung cancer, *OXA* oxaliplatin, *PTX* paclitaxel, *VCR* vincristine

### Drug metabolism enzymes-mediated herb-drug interactions

ACDs undergo phase I and/or phase II metabolic reactions in the body, producing inactive or active metabolites. Among all phase I drug-metabolizing enzymes, cytochrome P450 enzymes (CYPs) play a key role in the metabolism of ACDs. In particular, CYP3A4, CYP2D6, CYP1A2, and CYP2C8 are the main CYP isoenzymes involved in ACDs metabolism. Investigations by Gougis et al. indicated that approximately 50% of anticancer drugs are metabolized via CYP3A4 [2]. Many herbal medicines could either induce or inhibit CYP3A4 activity, which is the most frequently reported cause of PK-mediated HDIs.

Induction of CYP3A4 activity enhances the metabolism of substrate drugs, leading to decreased AUC and C_max_, which often results in reduced therapeutic efficacy and lower toxicity. St. John’s Wort (SJW) is a typical inducer of CYP3A4 and CYP2B6, with its main active compound, hyperforin, increasing CYP3A4 activity via activation of the pregnane X receptor [94]. Clinical practice has long proven that SJW reduces the bioavailability, C_max_, and half-life of imatinib (IM), irinotecan (CPT-11), and DTX by inhibiting CYP3A4, reducing their efficacy and ADR [95]. In contrast, inhibition of CYP3A4 activity can increase AUC to substrate drugs, which may lead to severe safety concerns, particularly for ACDs with a narrow therapeutic window. For instance, *Panax ginseng* has been shown to inhibit the CYP3A4 metabolism of IM in humans, ultimately causing hepatotoxicity in chronic myeloid leukemia patients; the hepatotoxic reactions diminished after discontinuation of *Panax ginseng* [96]. Similarly, a recent case report described a female metastatic breast cancer patient developed grade III ribociclib-induced liver injury, which was caused by the *Trigonella foenumgraecum* L.’s CYP3A4 inhibition, and hepatotoxicity improved after stopping supplement [19]. In addition, CYP3A4-mediated interactions were also observed in a male NSCLC cancer patient treated with echinacea and etoposide, resulting in severe thrombocytopenia [97].

In addition to CYP-mediated phase I metabolism, many ACDs undergo phase II conjugation reactions, in which drug molecules combine with endogenous substances such as glucuronic acid or sulfate to form highly polar inactive metabolites for excretion. UDP-glucuronosyltransferases (UGTs) are the most important enzymes in phase II conjugation reactions, mediating approximately 35% of phase II metabolism [98]. UGTs are the largest detoxification enzymes in the body. Many herbal products rich in flavonoids, lignans, and anthraquinones have been found to inhibit UGTs [99]. Co-administration of such herbal medicines with UGT substrate drugs may result in severe safety issues. CPT-11 is converted by carboxylesterases into its active metabolite, SN-38, which induces gastrointestinal toxicity through mucosal damage. Hepatic UGTs (UGT1A1 and UGT1A9) detoxify SN-38 by converting it into its inactive glucuronide metabolite. Thus, co-administration of herbal medicines that inhibit UGT1A1 and UGT1A9 is risky [100, 101]. Studies have shown that baicalein (extracted from *Scutellaria baicalensis* Georgi) competitively inhibits the glucuronidation of SN-38 by inhibiting UGT1A1 [102]. Zhang et al. also demonstrated significant HDIs between CPT-11 and herbal medicines containing psoralen or flavonols, resulting in increased gastrointestinal toxicity of CPT-11 [103]. Additionally, UGT1A1 is responsible for bilirubin metabolism in the body, and inhibition of UGT1A1 activity by herbal medicines may lead to hyperbilirubinemia, especially when co-administered with UGT1A1-inhibiting ACDs (e.g. SOF). Multiple studies have suggested that the concurrent use of SOF with UGT1A1 herbal inhibitors should be avoided [104]. Reports on the induction of UGTs by herbal medicines are relatively limited. Increased UGT activity can enhance detoxification, thereby reducing the ADR of ACDs. SJW accelerates the glucuronidation of SN-38, significantly reducing hematological and gastrointestinal toxicity. This effect may be attributed to decreased SN-38 exposure levels, as well as the anti-inflammatory and anti-apoptotic properties of SJW [105–107].

Other Phase II metabolizing enzymes include glutathione S-transferase (GST), sulfotransferases (SULTs), catechol-O-methyltransferases (COMTs), and N-acetyltransferases (NATs) [23]. Although research on these metabolizing enzymes in HDIs is limited, their importance should be underscored. For example, GST catalyzes the conjugation of glutathione with ACDs, therefore reducing their efficacy [27]. Oridonin overcomes PANC-1/Gem cells gemcitabine resistance by inhibiting GST metabolic activity [108].

### Drug transporters-mediated herb-drug interactions

DTs are transmembrane proteins widely expressed in various tissues and organs, playing a critical role in drug absorption, distribution, metabolism, and excretion. Transporters represent another key factor influencing drug concentrations in the human body. In recent years, an increasing number of clinical and basic studies have emphasized the significant role of transporters in the occurrence of HDIs. Numerous ACDs and their metabolites have been identified as substrates of DTs, and herbal medicines can modulate drug concentrations in the blood, tissues, or even within tumors by inhibiting or inducing the function and expression of transporters. DTs can be classified into uptake transporters and efflux transporters based on their transport direction.

Uptake transporters, including organic anion transporting polypeptides (OATPs), organic anion transporters (OATs), and organic cation transporters (OCTs), belong to the solute carrier transporters family. These transporters are widely distributed in various tissues and organs, such as the liver, kidneys, and intestines. They are primarily responsible for the uptake of small-molecule drugs and endogenous substances. They mediate the process of transporting substrates from the extracellular environment (typically the blood circulation) into the intracellular via passive diffusion or secondary active transport.

Many herbs or natural products have been reported to inhibit the function or expression of uptake transporters. This inhibition often prevents drug molecules from entering non-target organs such as the kidneys and liver, thereby reducing the organ toxicity of ACDs. For example, CDDP and MTX are taken up into renal epithelial cells through OCTs located on the basolateral membrane of renal tubules. The drug molecule accumulation in the kidney induces oxidative stress and ROS production, leading to proximal tubular necrosis and acute kidney injury. Natural products such as 6-methoxyflavone and wedelolactone have been shown to protect the kidneys of mice by inhibiting OCT2-mediated CDDP uptake [109, 110]. Similarly, Wang et al. reported that isosinensetin attenuated MTX-induced nephrotoxicity by inhibiting OCT3 uptake function and reducing MTX renal concentrations [111]. In the basolateral membrane of hepatocytes, OATPs mediate the uptake of various ACDs into liver cells. Inhibiting OATP activity can similarly reduce the ADR of hepatotoxic ACDs. Natural products luteolin have been shown to mitigate MTX-induced cytotoxicity by inhibiting the transport activity of OATP1B1 [112].

Efflux transporters, including P-glycoprotein (P-gp), breast cancer resistance protein (BCRP), multidrug resistance proteins (MRPs), etc., are primarily members of the ATP-binding cassette transporters family. These proteins are expressed on the apical membranes of many secretory cell types, such as intestines, liver, kidneys, adrenal glands, and physiological barriers, including the blood–brain barrier (BBB) and blood-testis barrier. They actively transport substrates out of cells, performing a detoxification function by excreting substances into the intestinal lumen, bile canaliculi, and renal tubules, thereby protecting the body from exogenous compounds.

Inhibition of efflux transporters by herbal medicines influences the bioavailability of ACDs, increases drug concentrations in tumors or blood circulation, and ultimately enhances therapeutic efficacy. However, this is always accompanied by increased toxicity. P-gp and BCRP are highly expressed in intestinal epithelial cells, and their inhibition by herbal products can increase the oral bioavailability of ACDs. Boonnop et al. found that black ginger extract and its active compound, 5,7-dimethoxyflavone, enhanced the oral absorption of PTX by inhibiting P-gp and BCRP [113]. Similarly, Korean red ginseng extracts increased PTX bioavailability by inhibiting P-gp, thereby enhancing PTX’s anticancer effects against breast cancer [114]. In physiological barriers such as the BBB, P-gp and BCRP are highly expressed and efflux exogenous toxins, exhibiting neuroprotective effects. However, this also limits the entry of therapeutic drugs into the brain, diminishing efficacy. Numerous studies have focused on inhibiting one or both of these transporters to prolong the mean residence time of ACDs in the brain. For instance, the natural product botryllamide G, a potent BCRP inhibitor, when combined with tariquidar (a P-gp inhibitor), nearly doubled the brain exposure of lapatinib in mice [115]. Beyond normal physiological tissues, efflux transporters are also highly expressed in various cancer cells. Efflux transporters actively expel ACDs and prevent intracellular drug accumulation in cancer cells, thus contributing to the development of MDR. Herbal medicines can inhibit P-gp, BCRP, or MRPs, thereby reversing tumor MDR. This is particularly relevant for cytotoxic ACDs, such as DOX, PTX, and vincristine (VCR). Recent studies have demonstrated that various herbal medicines, such as *Scabiosa atropurpurea L.*, inhibit the function of P-gp or downregulate its expression. These interventions have increased DOX accumulation and enhanced its antitumor activity in drug-resistant cancer cell lines (e.g., A2780/DX, LoVo/DX) [116–123]. On the other hand, increased DOX exposure also exacerbates side effects. *Dioscorea bulbifera* L. has been reported to delay DOX excretion by inhibiting P-gp, leading to worsening cardiac and renal toxicity and decreased survival rates [18].

In contrast, studies regarding herb-induced transporter activity are few and mainly associated with SJW. Low doses of SJW have been shown to induce intestinal P-gp expression in clinical practice, reducing drug absorption and consequently decreasing the bioavailability and therapeutic efficacy of co-administered drugs [124]. However, SJW does not appear to induce P-gp expression at the BBB. Therefore, co-administration of SJW with P-gp substrate drugs targeting the central nervous system is not expected to result in HDIs [125].

It is worth noting that genetic polymorphisms in DME or DT are likely to influence the extent of interactions with herbal medicines. Compared to individuals with weak metabolic or transport phenotypes, those with strong phenotypes are more susceptible to inhibition by DME or DT, and the degree of inhibition is greater [126]. For example, *Radix Astragali* had no statistically significant effect on the C_max_ and AUC of fexofenadine in *ABCB1 3435T* carriers, but it significantly prolonged the half-life in individuals with the *ABCB1 345CC* genotype [127]. Similarly, garlic reduces the systemic clearance of DTX in patients with the CYP3A5 expressive phenotype (CYP3A5*1/*1), but this effect was not observed in patients with the non-expressive phenotype (CYP3A5*3/*3) [128]. It is important to note that the impact of genetic polymorphisms on HDIs has not been as thoroughly characterized as DDIs. The mechanisms underlying the association between genotype and HDIs in oncology treatment require further research.

## Limitation of current herb-drug interactions studies

### Unknown chemical composition

Unlike most pharmaceutical products with well-defined chemical structures and a robust foundation of pharmacological research, herbal medicines are typically composed of multiple chemical constituents. The net effect of the interaction could contribute to the additive effects of these individual components. Thus, merely studying HDIs between whole herbal preparations and ACDs is insufficient. It is essential to analyze the whole chemical constituents of the herbs, evaluate their effects individually, and identify the causative chemical constituents (CCCs) that contribute to the HDIs. This approach can provide a reference for HDI studies involving other herb medicines with similar CCCs structure. However, limited herbal medicines, such as SJW and milk thistle, have been fully characterized by chemical composition. Many HDIs studies are confined to herbal extracts or focus on a single major constituent of the herb, resulting in research bias. Consequently, the findings of such studies often have limited research value and fail to establish correlations between chemical structures and the occurrence of HDIs. HDI researchers are encouraged to foster interdisciplinary collaborations in fields such as medicinal chemistry, ensuring that the results are more scientifically robust and valuable.

### Variability in herbal component

For herb medicine characterized by chemical composition, this composition is not fixed. The chemical constituents of the same herb may vary significantly depending on the place of origin. This variability often renders research findings of HDIs for the same herb non-generalizable. For example, the use of ginseng (250 mg/day) in NSCLC patients induced CYP3A4 activity, thereby increasing the metabolism of gefitinib and reducing efficacy [129]. However, this finding contradicts the CYP3A4 inhibitory effect observed in the co-administration of ginseng with IM. Audrey et al. attributed this variability to differences in the ginsenoside content of commercial ginseng preparations [130, 131]. In addition, different extraction methods also lead to variability in chemical composition, ultimately affecting research outcomes. Benkovic et al. reported that the combination of ethanol or water extracts of propolis with irinotecan significantly extended the median survival time of tumor-bearing mice [132]. However, only the ethanol extract exhibited a significantly synergistic antitumor effect. Research indicates that the total flavonoid and polyphenol contents in the ethanol extract of propolis were significantly higher than those in the water extract, which might explain the stronger anticancer activity of the ethanol extract in combination therapy [133]. Therefore, in research, it is crucial to meticulously document the manufacturer, origin, batch number, preparation method, and chemical composition of the herbs to ensure reproducibility of the results and facilitate comparison with other studies.

## Risk evaluation strategy in herb-drug interactions

Numerous investigations on the mechanisms of HDIs focus solely on either PK or PD aspects, rendering their conclusions less applicable to clinical practice, particularly for ACDs that could benefit from synergy effects. For instance, oridonin has been found to enhance the mRNA expression of various CYP enzyme families (1a, 2a, 2d, 2e, 2c, and 3a) and induce CYP enzyme activity. Zhang and colleagues suggested that the clinical application of oridonin might pose potential risks for HDIs [134]. However, subsequent studies revealed that oridonin exhibits synergy effects with ACDs in various cancer cells, likely due to its anti-angiogenic effect and inhibition of the anti-apoptotic protein Bax-2 [52, 135]. Due to SJW’s strong CYP3A4-inducing effect, early studies have cautioned against the co-administration of SJW with medications. However, Chen et al. found that hypericin (extracted from SJW) synergized with DOX to kill MDA-MB-231 cells and alleviated DOX-induced cardiac toxicity [84], suggesting the potential benefits of combination therapy. This bias is primarily due to the complexity arising from the multi-component of herbal medicines and their multi-target effects when combined with ACDs. Judging the compatibility of a herb with ACDs based solely on one aspect of research is overly simplistic and risks overlooking a promising ACD sensitizer. HDIs-related research should be conducted from a holistic and dialectical perspective.

In recent years, advancements in computer technology have led to the broth of various DDI risk prediction methods in silico. These methods quantitatively predict the net effect of combination therapy by calculating the effects of drugs on DME, DT, and targets. This research method, which integrates multidimensional information from PK and PD, is particularly well-suited for the complex study of HDIs. Therefore, we further summarize the current comprehensive risk prediction strategies to assess the suitability of combining herbs with ACDs from a holistic, systemic perspective.

### Modelling and simulation approaches

Modelling and Simulation (MS) approaches are emerging computational techniques that abstract key factors influencing drug disposition and pharmacological effects through various quantitative methods. These approaches establish mathematical models and integrate specific information about exogenous substances to predict potential drug-drug interactions (DDIs), support decision-making in drug development, and optimize clinical treatment regimens. Since 2012, regulatory agencies such as the FDA and EMA have officially recognized the pivotal role of MS approaches in predicting the risk of DDIs during new drug development and have issued corresponding regulatory guidance [136, 137]. Notably, physiologically based pharmacokinetic (PBPK) modelling is particularly emphasized in these guidelines for its ability to predict the risk of DDIs.

The PBPK model can forecast the effects of perpetrator compounds on drug-metabolizing enzymes and transporters, enabling the prediction of potential clinical DDIs. Establishing a PBPK model requires two sets of parameters: physiological parameters (e.g., organ weight, organ blood flow rate) can be obtained from literature, while compound-specific parameters (e.g., tissue partition coefficients, absorption rate constants, and metabolic clearance rates) need to be determined through in vitro, in vivo studies, or clinical trials. These parameters can also be estimated based on the physicochemical properties of the respective compounds. PBPK models for both victim and perpetrator compounds can be linked through appropriate interaction mechanisms, such as reversible inhibition (RI) or time-dependent inhibition (TDI), to simulate PK-mediated DDIs [136]. Various commercial software platforms are available to facilitate PBPK model development. Differential equation-solving software packages include MATLAB Simulink, Berkeley Madonna, Wolfram Mathematica, and acslX. These programs do not contain predefined model structures or differential equations, making the complexity and flexibility of the model dependent on the researcher’s objectives and programming capabilities. On the other hand, software such as Simcyp, PK-Sim, GastroPlus, and MATLAB SimBiology provide template-based model structures, although at the cost of complete customization [138].

Although PBPK model-based predictions of DDIs are well-established, research on predicting HDIs remains relatively limited. Current studies mainly focus on herbs or natural products with well-defined clinical PK profiles, such as SJW, milk thistle, and Wuzhi capsules. Furthermore, existing research predominantly centres on the effects of herbs on DME.

Pilla Reddy et al. developed a PBPK-based HDI risks decision tree by integrating in vitro and clinical PK data of the main components of SJW-bergamottin, curcumin, and hyperforin. This model successfully predicted a moderate clinical HDI risks (1.57-fold) between SJW and anticancer drugs such as acalabrutinib, osimertinib, and olaparib [139]. Similarly, Adiwidjaja et al. established a PBPK model for hyperforin to predict the effects of SJW on CYPs and evaluated interactions between hyperforin and substrates of CYP3A, CYP2C9, and CYP2C19. Their study revealed that hyperforin concentrations are significantly higher in the intestine compared to the liver, with intestinal CYPs induction being more pronounced than hepatic CYPs induction (15.5-fold vs. 1.1-fold, respectively) [140].

Gufford et al. developed a quantitative model to assess the risk of clinical interactions between silybin, the main active ingredient of milk thistle, and raloxifene. The model result indicated that silybin could increase the AUC and C_max_ of raloxifene by 30% by affecting intestinal glucuronidation [141]. This predictive modelling was further validated in a clinical trial, where silybin caused a 9% increase in the AUC of raloxifene in healthy volunteers, with one subject experiencing a twofold increase in AUC and a threefold increase in C_max_., further demonstrating the potential applicability of PBPK modelling in predicting clinical HDI risks.

The main active ingredient of Wuzhi capsules is extracted from *Schisandra sphenanthera plant* (Magnoliaceae family), which has hepatoprotective effects and is clinically approved to be used in combination with the immunosuppressant Cyclosporin A (CsA) to alleviate CsA-induced hepatotoxicity. Fan et al. used PBPK modelling to predict that multiple doses of two major active components of Wuzhi capsules, schisandrin A and schisandrol B, could increase CsA’s AUC by 226 and 36%, respectively. This suggests that when combined clinically, CsA doses can be reduced to lower the risks of ADR [142]. Multiple doses of schisandrin A and schisandrol B were also predicted to increase the AUC of MTX by 29 and 301%, and C_max_ by 7 and 75%, respectively [143]. He et al.’s PBPK model predicted that schisantherin A from Wuzhi capsules exhibited TDI of CYP3A4, while schisantherin B exhibited both RI and TDI of CYP3A4 and CYP3A5. Multiple doses of schisantherin B were predicted to increase the AUC of tacrolimus by 26% in CYP3A5 expressers and by 57% in non-expressers [144]. Additionally, Adiwidjaja’ PBPK model predicted that schisandrol B and schisantherin B could effectively inhibit CYP3A4-mediated metabolism of IM and bosutinib. Co-administration in clinical practice could increase bosutinib exposure (AUC ratio of 3.0), but would not affect IM exposure [145]. These PBPK models collectively provide a framework for the prospective evaluation of HDI potential, offering evidence-based insights into the risks or safety of herb-drug combinations.

### Herb-drug interactions database

Herb-drug interactions database is a structured data collection system designed to rapidly retrieve critical HDI risks extracted from a large body of literature, providing essential information for clinical practitioners. Since the public became aware of the clinical significance of HDIs, researchers have been attempting to establish HDIs databases using various information technologies as early as the 1990s [146–148]. Those databases enhance clinical decision-making by systematically compiling scientific evidence of HDIs, providing risks assessment management strategies to enable clinical practitioners avoid ADR in herb-drug combinations. More importantly, the categorical compilation of HDI mechanisms of action allows for rapid identification of interaction characteristics and thus prediction of unobserved interactions [149].

To date, more than ten free or commercial HDIs databases have been developed [150]. Among the free databases, prominent examples include the Chi Mei Search System (CMSS), the Chinese-Western Medicine Integrative Information Network (CWMIN), the Drug Herb Interaction Query Website (DHIQW), the Center of Excellence for Natural Product-Drug Interaction Research, and the Probot Chinese Medicine-Drug Interaction Database. Commercial databases primarily include the UW Drug Interaction Database (DIDB), Hédrine, Lexicomp Drug Interactions, the Natural Medicines Comprehensive Database, and Stockley’s Herbal Medicines Interactions.

As research on HDIs progresses, these databases require regular updates, with update frequencies ranging from daily to annually. However, HDIs information is often buried in various textual sources such as research papers, conference reports, books, and drug evaluation reports, making structured data extraction highly challenging [150]. This process requires a combination of extensive medical research expertise and strong information technologies support. Due to a lack of sustained funding, most free databases (e.g., CMSS, CWMIN, and DHIQW) have stopped receiving updates after their initial release. In contrast, commercial databases have continued to be maintained and updated. Among them, DIDB is currently the largest and is updated daily. Established in 2002 by Dr. Renée Levy at the University of Washington [151], DIDB contains the most extensive manually curated in vitro and clinical data, encompassing interaction events under various conditions, including co-administered drugs, excipients, food products, herbal medicines, tobacco, organ damage, and genetic factors. This database integrates information from literature, drug labels, FDA new drug applications, and biologics license applications. Through manual extraction, the data are presented in a structured format based on their underlying mechanisms within the DIDB [149, 152]. Interaction outcomes in DIDB include not only PK but also PD and safety data. As of June 2021, the application contained a total of 2,539 natural products (including herbal and food products) and 15,864 drug interaction experiments/studies [150].

Despite the advancements represented by HDIs databases, significant limitations persist. Poor consistency of predictive results across databases [153]**.** A study using Hédrine and the MSKCC database to prospectively assess the occurrence of HDIs in outpatients taking oral ACDs identified 46 HDIs in Hédrine and 22 in the MSKCC database*,* with only 9.5% of interactions common to both [5]. This low concordance can be explained by the absence of certain herbal medicine in one or the other database. In addition, high maintenance costs and non-standardised herb nomenclature likewise contribute to the limitations of the database risks assessment [150]. Future advancements require expanding data coverage and integrating AI models to address complex therapeutic scenarios globally.

### Network pharmacology

Network pharmacology based on systems biology, genomics, proteomics, and other disciplines has emerged recently. It utilizes omics data analysis and computer simulation technologies to reveal the network relationship of drug-gene-target-disease interactions, and predicts the action mechanism, drug efficacy and ADR. Network pharmacology’s holistic, systematic and complete nature makes it very suitable for studying herbal medicine with multiple components and multitargets [154, 155]. In particular, the gene targets screening of tumor and drug metabolism has made a unique contribution in predicting HDI risks and explaining the mechanism of PD/PK interaction. Herb pair of *Radix Astragali* and *Rhizoma Curcumae Phaeocaulis* (HQEZ) has been found to reduce toxicity and increase the therapeutic effect of 5-FU [156]. Network analysis of HQEZ revealed that the 4 core compounds (folate, curcumin, quercetin and kaempferol) could affect chemoresistance and 5-FU sensitivity related targets, such as AKT1, EGFR, P-gp, BCRP, MMP2, TLR4, TLR9 and so on [157]. Systems pharmacology screening of *Phyllanthus fraternus* (PF) predicted 51 genes related to drug metabolism or drug transport, including ABCB1, CYP1A1, CYP1A2, CYP2C9, and CYP3A4, suggesting potential HDIs interactions [158]. Molecular docking and molecular dynamics further demonstrated that the three core components (2,4-bis(1,1-dimethylethyl)-phenol, 5-mecyloxy-*N*-[(5-methylpyridin-2-yl)sulphonyl]-1*H*-indole-2-carboxamide and E, E. Z-1,3,12-ninene-5,14-diol) interact more with the target.

Although the existing network pharmacology has been well used in predicting the mechanism of action of herbal medicines and the risks of HDIs. However, insufficient reliable data on herbal compounds remains an obvious obstacle to constructing sufficiently predictable herbal networks [159]. Another important challenge lies in the scientific and reliable validation of predictive results. The integration of technologies such as artificial intelligence is expected to bridge the network pharmacology limitations and strengthen the predictive reliability of herbal networks.

### Machine learning and artificial intelligence

In recent years, with the development of artificial intelligence, researchers have tried to use machine learning (ML) to predict risks of drug interactions in clinical practice. ML leverages complex algorithms and mathematical methods to cluster and normalize large datasets, followed by calculations and predictions from large-scale omics data. Tools such as BestComboScore [160], DrugComb [161], DrugComboRanker [162], and DeepSynergy [163] have been used to predict drug combinations by employing deep learning (DL) and statistical models. These ML-based tools for drug combinations are of great significance in understanding HDIs.

The development of ML-based HDIs prediction models requires two types of information: biological (e.g., synergy datasets from herb-drug combination matrices, gene expression, microRNA expression, and proteome) and chemical (e.g., chemical fingerprints and molecular descriptors of herbal products) [154]. However, due to the lack of comprehensive material basis information, variability in the chemical composition of herbal medicines, and other related factors, ML-based HDIs prediction remains relatively underdeveloped and requires further advancement.

In addition, AI technology has been applied to the development of databases, as exemplified by SUPP.AI, which is currently the only HDIs database utilizing AI technology. In 2019, a team from the Allen Institute for Artificial Intelligence (AI2) developed the SUPP.AI database to identify and catalog supplement-drug interactions, including those involving herbal medicines, using machine learning and natural language processing (NLP) techniques [164]. By leveraging AI, SUPP.AI can automatically extract HDIs information from biomedical literature, significantly reducing labor costs and enabling the database to remain freely accessible and regularly updated. As of November 2024, SUPP.AI had recorded approximately 2044 supplements, 2866 drugs, and 59,096 interactions [165]. Researchers and clinicians can search the database using keywords such as the name of an herb or drug. Search results include potential HDIs outcomes on entity pages, as well as related evidence sentences on interaction pages, where herbs and drugs are highlighted in each sentence. The evidence sentences are accompanied by their sources, with further source details linked through the semantic scholar tool. However, challenges such as the ambiguous differentiation between drugs and herbs, the lack of standardized terminology for herbal products, and limitations in NLP technology seem to restrict SUPP.AI’s ability to identify potential HDIs information from the literature [164].

ML promotes data-driven decision-making, which is highly compatible with the multi-level data integration approach of network pharmacology. Although there is currently no successful practical application, several recent reviews have proposed the concept of combining ML with network pharmacology and elucidated its underlying logic [166–168]. ML methods such as support vector machines, random forests, and DL have been proposed to enhance decision-making levels and accuracy of network pharmacology [169–171]. In this context, network pharmacology will benefit from ML-driven artificial intelligence models to represent drug-target, herb-target, drug-pathway, and herb-pathway interactions; supervised learning for interaction prediction; generative modelling; and hybrid models for integrating various data sources [168]. Particularly for herbs, this can encompass all known compounds with any pharmacological effects. This approach not only identifies direct HDIs but also secondary interactions that may arise due to downstream effects of metabolism or signaling pathways. In this process, obtaining high-quality, manually verified datasets to train ML models is currently an urgent challenge that needs to be addressed [166].

## Summaries and future perspectives

TCM physicians have recognized the drugs interactions, leading to the creation of multi-herb prescriptions to achieve synergy or detoxification effect [23]. For example, there are prescriptions designed to enhance the efficacy of herbal medicines based on compatible TCM theories ‘Monarch, Minister, Assistant, and Guide’, as well as prohibited combinations based on “Nineteen Medicaments of Mutual Antagonism” [172, 173]. These ancient theories of TCM are rooted in extensive clinical practice, indicating that the paradigm of drug combinations has a long history. In recent years, the medical community has increasingly recognized the importance of drug interactions, shifting focus from DDIs to an increasing number of reported HDIs. This reflects a growing awareness of the prevalence of herbal medicines and the significant clinical benefits/risks associated with HDIs, particularly in oncology treatment. Early researchers generally regarded HDIs as adverse events, believing that herbs could affect the efficacy of ACDs, leading to unpredictable clinical outcomes. However, with the advancement of HDIs research, increasing evidence suggests that the combination of herbs and ACDs may provide beneficial effects for cancer patients. Nevertheless, due to the lack of comprehensive elucidation of HDIs mechanisms, combining herbal medicines and ACDs remains a topic of considerable debate.

Although modelling simulations, databases, network pharmacology and machine learning tools are widely used in drug discovery and have found some applications in herbal medicines research, their application in predicting HDIs is still in its early stages. For example, while PBPK models for predicting DDIs are well-established, HDIs predictions remain relatively underdeveloped. This is largely due to the complexity of herbal compositions, the lack of identification of CCCs, and insufficient clinical PK data for critical compounds. Furthermore, much of the existing research centres on the effects of herbs on DME, with relatively little attention to other potential mechanisms of HDIs. Additionally, challenges such as the unclear composition of many herbal medicines, the lack of standardized terminology for herbal products, and limitations in NLP technology significantly hinder the development of comprehensive and accurate HDIs databases. These issues complicate the extraction and organization of reliable data from literature, which is crucial for constructing databases that can accurately predict herb-drug interactions. Advancing this field requires collaborative efforts between systems biology experts, bioinformaticians, AI researchers, and herbal medicines specialists. Such interdisciplinary cooperation is essential to overcoming current challenges and accelerating progress in HDIs research, ultimately leading to more accurate predictions and safer clinical applications.

In clinical oncology, based on the risk prediction methods, we believe that some clinical strategies can be practiced to reduce the risks associated with HDIs. First, pre-treatment risk assessment. Prioritize herbs with established PK/PD profiles and avoid combinations with narrow therapeutic index ACDs (e.g., CPT-11, IM) using modelling simulations, HDIs databases, etc. Second, during-treatment endogenous biomarkers monitoring. Since the main physiological role of DME and DT is to dispose of various endogenous compounds, some studies in recent years have proposed to characterize the activity of DME and DT by detecting changes in the levels of these biomarkers, which has been well applied in the early DDI studies of drug discovery [174]. The real-time monitoring biomarkers during the course of herb-drug combination therapy can timely terminate the occurrence of ADR, which is even more promising in ACDs mostly metabolized by CYP3A4. Finally, dynamic dose adjustment, using AI algorithms to integrate real-world data to improve the combination drug delivery scheme, to achieve individualized and precise oncology treatment [175].

## Conclusion

In conclusion, this review systematically analyzes the PD/PK mechanisms by which herbs influence ACDs and summarizes current predict methods for HDI risks, aiming to promote these predictive approaches to address existing limitations in HDIs research. Finally, we propose clinical mitigation frameworks to harness the therapeutic potential of herb-ACD combinations while minimizing risks in precision oncology.

## Data Availability

Not applicable.

## Abbreviations

TCMs: Traditional Chinese Medicines
HDIs: Herb–drug interactions
ACDs: Anticancer drugs
MDR: Multidrug resistance
PK: Pharmacokinetic
PD: Pharmacodynamic
C_max_: Blood peak concentration
AUC: Area under curve
DME: Drug metabolizing enzymes
DT: Drug transporters
CYPs: Cytochrome P450 enzymes
SJW: St. John’s Wort
IM: Imatinib
CPT-11: Irinotecan
DTX: Docetaxel
UGTs: UDP-glucuronosyltransferases
SOF: Sorafenib
OATPs: Organic anion transporting polypeptides
OATs: Organic anion transporters
OCTs: Organic cation transporters
CDDP: Cisplatin
MTX: Methotrexate
ROS: Reactive oxygen species
P-gp: P-glycoprotein
BCRP: Breast cancer resistance protein
MRPs: Multidrug resistance proteins
BBB: Blood-brain barrier
PTX: Paclitaxel
DOX: Doxorubicin
VCR: Vincristine
5-FU: 5-Fluorouracil
CTX: Cyclophosphamide
Ara-C: Cytarabine
GFT: Gefitinib
EGCG: (−)-Epigallocatechin gallate
BZM: Bortezomib
EMT: Epithelial-mesenchymal transition
PCD: Programmed cell death
TNBC: Triple-negative breast cancer
HCC: Hepatocellular carcinoma
FA: Fanconi anemia pathway
NSCLC: Non-small cell lung cancer
CSCs: Cancer stem cells
GC: Gastric carcinoma
AKI: Acute kidney injury
NS: Nephrotic syndrome
CIPN: Chemotherapy-induced peripheral neurotoxicity
OXA: Oxaliplatin
GJG: Goshajinkigan
CTX: Cyclophosphamide
TPT: Topotecan
MMC: Mitomycin C
TMZ: Temozolomide
MS: Modelling and simulation
DDIs: Drug–drug interactions
PBPK: Physiologically based pharmacokinetic
RI: Reversible inhibition
TDI: Time-dependent inhibition
CsA: Cyclosporin A
IT: Information technologies
CMSS: Chi Mei search system
CWMIN: Chinese-Western Medicine Integrative Information Network
DHIQW: Drug Herb Interaction Query Website
AI: Artificial intelligence
ML: Machine learning
NLP: Natural language processing
ORR: Objective response rate
DCR: Disease control rate
QOL: Quality of life

## References

[1] Rashrash M, Schommer JC, Brown LM. Prevalence and predictors of herbal medicine use among adults in the United States. J Patient Exp. 2017;4(3):108–13. 28959715 10.1177/2374373517706612 PMC5593261

[2] Gougis P, Hilmi M, Geraud A, Mir O, Funck-Brentano C. Potential cytochrome P450-mediated pharmacokinetic interactions between herbs, food, and dietary supplements and cancer treatments. Crit Rev Oncol Hematol. 2021;166: 103342. 33930533 10.1016/j.critrevonc.2021.103342

[3] National Health Commission of the People’s Republic of China. National Essential Medicines List (2018 edition). Beijing: NHC; 2018.

[4] Kumar S, Mittal A, Babu D, Mittal A. Herbal medicines for diabetes management and its secondary complications. Curr Diabetes Rev. 2021;17(4):437–56. 33143632 10.2174/1573399816666201103143225

[5] Prely H, Herledan C, Caffin AG, Baudouin A, Larbre V, Maire M, et al. Real-life drug-drug and herb-drug interactions in outpatients taking oral anticancer drugs: comparison with databases. J Cancer Res Clin Oncol. 2022;148(3):707–18. 33914124 10.1007/s00432-021-03645-z PMC11800911

[6] Zhou X, Chan K, Yeung JH. Herb-drug interactions with Danshen (*Salvia miltiorrhiza*): a review on the role of cytochrome P450 enzymes. Drug Metabol Drug Interact. 2012;27(1):9–18. 22718621 10.1515/dmdi-2011-0038

[7] Stoddard GJ, Archer M, Shane-McWhorter L, Bray BE, Redd DF, Proulx J, et al. Ginkgo and warfarin interaction in a large veterans administration population. AMIA Annu Symp Proc. 2015;2015:1174–83. 26958257 PMC4765589

[8] Biswas S, Mahapatra E, Ghosh A, Das S, Roy M, Mukherjee S. Curcumin rescues doxorubicin responsiveness via regulating aurora a signaling network in breast cancer cells. Asian Pac J Cancer Prev. 2021;22(3):957–70. 33773562 10.31557/APJCP.2021.22.3.957 PMC8286672

[9] Zhou Y, Zhou C, Zou Y, Jin Y, Han S, Liu Q, et al. Multi pH-sensitive polymer-drug conjugate mixed micelles for efficient co-delivery of doxorubicin and curcumin to synergistically suppress tumor metastasis. Biomater Sci. 2020;8(18):5029–46. 32812957 10.1039/d0bm00840k

[10] Younes M, Mardirossian R, Rizk L, Fazlian T, Khairallah JP, Sleiman C, et al. The synergistic effects of curcumin and chemotherapeutic drugs in inhibiting metastatic, invasive and proliferative pathways. Plants (Basel). 2022;11(16):2137. 36015440 10.3390/plants11162137 PMC9414747

[11] Wattanapitayakul SK, Chularojmontri L, Herunsalee A, Charuchongkolwongse S, Niumsakul S, Bauer JA. Screening of antioxidants from medicinal plants for cardioprotective effect against doxorubicin toxicity. Basic Clin Pharmacol Toxicol. 2005;96(1):80–7. 15667600 10.1111/j.1742-7843.2005.pto960112.x

[12] Russo ER, Facincani I, Nakazato KC, Coimbra TM, Crevelin EJ, Pereira AMS, et al. Oral administration of powdered dried rhizomes of *Curcuma longa* L. (turmeric, Zingiberaceae) is effective in the treatment of doxorubicin-induced kidney injury in rats. Phytother Res. 2018;32(12):2408–16. 30109739 10.1002/ptr.6176

[13] Fan HY, Wang XK, Li X, Ji K, Du SH, Liu Y, et al. Curcumin, as a pleiotropic agent, improves doxorubicin-induced nephrotic syndrome in rats. J Ethnopharmacol. 2020;250: 112502. 31881321 10.1016/j.jep.2019.112502

[14] Milling L, Zhang Y, Irvine DJ. Delivering safer immunotherapies for cancer. Adv Drug Deliv Rev. 2017;114:79–101. 28545888 10.1016/j.addr.2017.05.011 PMC5647831

[15] Bazrafshani MS, Pardakhty A, Kalantari Khandani B, Tajadini H, Ghazanfari Pour S, Hashemi S, et al. The prevalence and predictors of herb-drug interactions among Iranian cancer patients during chemotherapy courses. BMC Complement Med Ther. 2023;23(1):41. 36750849 10.1186/s12906-023-03869-1 PMC9903537

[16] Lam CS, Koon HK, Ma CT, Au KY, Zuo Z, Chung VC, et al. Real-world data on herb-drug interactions in oncology: a scoping review of pharmacoepidemiological studies. Phytomedicine. 2022;103: 154247. 35716539 10.1016/j.phymed.2022.154247

[17] Ben-Arye E, Samuels N, Goldstein LH, Mutafoglu K, Omran S, Schiff E, et al. Potential risks associated with traditional herbal medicine use in cancer care: a study of Middle Eastern oncology health care professionals. Cancer. 2016;122(4):598–610. 26599199 10.1002/cncr.29796

[18] Qu X, Zhai J, Hu T, Gao H, Tao L, Zhang Y, et al. *Dioscorea bulbifera* L. delays the excretion of doxorubicin and aggravates doxorubicin-induced cardiotoxicity and nephrotoxicity by inhibiting the expression of P-glycoprotein in mice liver and kidney. Xenobiotica. 2019;49(9):1116–25. 29985077 10.1080/00498254.2018.1498560

[19] Al Harrak Y, Lkhoyaali S, Lamsyah O, Tine MM, Bechar H, Benabdallah G, et al. Ribociclib-Induced hepatotoxicity exacerbated by fenugreek supplement use: a case report. J Oncol Pharm Pract. 2025. 10.1177/10781552251340911. 40388649 10.1177/10781552251340911

[20] Bosch-Barrera J, Queralt B, Menendez JA. Targeting STAT3 with silibinin to improve cancer therapeutics. Cancer Treat Rev. 2017;58:61–9. 28686955 10.1016/j.ctrv.2017.06.003

[21] Xie Y, Wang C. Herb-drug interactions between Panax notoginseng or its biologically active compounds and therapeutic drugs: a comprehensive pharmacodynamic and pharmacokinetic review. J Ethnopharmacol. 2023;307: 116156. 36754189 10.1016/j.jep.2023.116156

[22] Zhou X, Fu L, Wang P, Yang L, Zhu X, Li CG. Drug-herb interactions between *Scutellaria baicalensis* and pharmaceutical drugs: Insights from experimental studies, mechanistic actions to clinical applications. Biomed Pharmacother. 2021;138: 111445. 33711551 10.1016/j.biopha.2021.111445

[23] Li M, Wang Y, Chen Y, Dong L, Liu J, Dong Y, et al. A comprehensive review on pharmacokinetic mechanism of herb-herb/drug interactions in Chinese herbal formula. Pharmacol Ther. 2024;264: 108728. 39389315 10.1016/j.pharmthera.2024.108728

[24] Niu J, Straubinger RM, Mager DE. Pharmacodynamic drug-drug interactions. Clin Pharmacol Ther. 2019;105(6):1395–406. 30912119 10.1002/cpt.1434 PMC6529235

[25] Abu Soukhon AA, Abu-Qatouseh L, Mansoor K, El-Hajji FD, Al-Najjar M, Awwad S, et al. Cytotoxicity activity of graviola fruit extract with carbamazepine and valproic acid show antagonistic and indifferent effects. Asian Pac J Cancer Prev. 2023;24(6):1869–75. 37378914 10.31557/APJCP.2023.24.6.1869 PMC10505864

[26] Popovich DG, Tiaras F, Yeo CR, Zhang W. Lovastatin interacts with natural products to influence cultured hepatocarcinoma cell (hep-g2) growth. J Am Coll Nutr. 2010;29(3):204–10. 20833993 10.1080/07315724.2010.10719835

[27] Guneidy RA, Gad AM, Zaki ER, Ibrahim FM, Shokeer A. Antioxidant or pro-oxidant and glutathione transferase P1–1 inhibiting activities for *Tamarindus indica* seeds and their cytotoxic effect on MCF-7 cancer cell line. J Genet Eng Biotechnol. 2020;18(1):74. 33215267 10.1186/s43141-020-00077-z PMC7677421

[28] Golden EB, Lam PY, Kardosh A, Gaffney KJ, Cadenas E, Louie SG, et al. Green tea polyphenols block the anticancer effects of bortezomib and other boronic acid-based proteasome inhibitors. Blood. 2009;113(23):5927–37. 19190249 10.1182/blood-2008-07-171389

[29] Pezzani R, Salehi B, Vitalini S, Iriti M, Zuniga FA, Sharifi-Rad J, et al. Synergistic effects of plant derivatives and conventional chemotherapeutic agents: an update on the cancer perspective. Medicina (Kaunas). 2019;55(4):110. 30999703 10.3390/medicina55040110 PMC6524059

[30] Nagaprashantha LD, Vatsyayan R, Singhal J, Fast S, Roby R, Awasthi S, et al. Anti-cancer effects of novel flavonoid vicenin-2 as a single agent and in synergistic combination with docetaxel in prostate cancer. Biochem Pharmacol. 2011;82(9):1100–9. 21803027 10.1016/j.bcp.2011.07.078 PMC3252753

[31] Peng F, Liao M, Qin R, Zhu S, Peng C, Fu L, et al. Regulated cell death (RCD) in cancer: key pathways and targeted therapies. Signal Transduct Target Ther. 2022;7(1):286. 35963853 10.1038/s41392-022-01110-y PMC9376115

[32] Gao W, Wang X, Zhou Y, Wang X, Yu Y. Autophagy, ferroptosis, pyroptosis, and necroptosis in tumor immunotherapy. Signal Transduct Target Ther. 2022;7(1):196. 35725836 10.1038/s41392-022-01046-3 PMC9208265

[33] Yang Y, Zhang Z, Li S, Ye X, Li X, He K. Synergy effects of herb extracts: pharmacokinetics and pharmacodynamic basis. Fitoterapia. 2014;92:133–47. 24177191 10.1016/j.fitote.2013.10.010

[34] Samandari-Bahraseman MR, Ismaili A, Esmaeili-Mahani S, Ebrahimie E, Loit E. *Bunium persicum* seeds extract in combination with vincristine mediates apoptosis in MCF-7 cells through regulation of involved genes and proteins expression. Anticancer Agents Med Chem. 2024;24(3):213–23. 38038013 10.2174/0118715206277444231124051035

[35] Song Q, Zhang J, Wu Q, Li G, Leung EL. Kanglaite injection plus fluorouracil-based chemotherapy on the reduction of adverse effects and improvement of clinical effectiveness in patients with advanced malignant tumors of the digestive tract: a meta-analysis of 20 RCTs following the PRISMA guidelines. Medicine (Baltimore). 2020;99(17): e19480. 32332600 10.1097/MD.0000000000019480 PMC7220674

[36] Liu Y, Zhang W, Wang XJ, Liu S. Antitumor effect of Kanglaite(R) injection in human pancreatic cancer xenografts. BMC Complement Altern Med. 2014;14:228. 25005526 10.1186/1472-6882-14-228 PMC4105135

[37] Zheng W, Zhou CY, Zhu XQ, Wang XJ, Li ZY, Chen XC, et al. Oridonin enhances the cytotoxicity of 5-FU in renal carcinoma cells by inducting necroptotic death. Biomed Pharmacother. 2018;106:175–82. 29958141 10.1016/j.biopha.2018.06.111

[38] Yang C, Lu T, Liu M, Yuan X, Li D, Zhang J, et al. Tiliroside targets TBK1 to induce ferroptosis and sensitize hepatocellular carcinoma to sorafenib. Phytomedicine. 2023;111: 154668. 36657316 10.1016/j.phymed.2023.154668

[39] Ferdousmakan S, Mansourian D, Seyedi Asl FS, Fathi Z, Maleki-Sheikhabadi F, Afjadi MN, et al. Autophagy induced by metabolic processes leads to solid tumor cell metastatic dormancy and recurrence. Med Oncol. 2025;42(3):62. 39899220 10.1007/s12032-025-02607-6

[40] Zhu Q, Zhang R, Gu X, Zhao Z, Gao Q, Chen M, et al. Honokiol enhances the sensitivity of cetuximab in KRAS(G13D) mutant colorectal cancer through destroying SNX3-retromer complex. Theranostics. 2024;14(14):5443–60. 39310106 10.7150/thno.97180 PMC11413778

[41] Zhang LD, Liu Z, Liu H, Ran DM, Guo JH, Jiang B, et al. Oridonin enhances the anticancer activity of NVP-BEZ235 against neuroblastoma cells in vitro and in vivo through autophagy. Int J Oncol. 2016;49(2):657–65. 27278249 10.3892/ijo.2016.3557

[42] Zhang N, Ng AS, Cai S, Li Q, Yang L, Kerr D. Novel therapeutic strategies: targeting epithelial-mesenchymal transition in colorectal cancer. Lancet Oncol. 2021;22(8):e358–68. 34339656 10.1016/S1470-2045(21)00343-0

[43] Xie W, Zhang Y, Zhang S, Wang F, Zhang K, Huang Y, et al. Oxymatrine enhanced anti-tumor effects of Bevacizumab against triple-negative breast cancer via abating Wnt/beta-Catenin signaling pathway. Am J Cancer Res. 2019;9(8):1796–814. 31497360 PMC6726986

[44] Chen Y, Chen S, Chen K, Ji L, Cui S. Magnolol and 5-fluorouracil synergy inhibition of metastasis of cervical cancer cells by targeting PI3K/AKT/mTOR and EMT pathways. Chin Herb Med. 2024;16(1):94–105. 38375055 10.1016/j.chmed.2023.01.004 PMC10874772

[45] Guo S, Li Y, Su H, Meng M, Xi J, Mo G, et al. Aidi injection as adjunctive treatment to gemcitabine-based chemotherapy for advanced non-small cell lung cancer: a systematic review and meta-analysis. Pharm Biol. 2021;59(1):1260–75. 34541998 10.1080/13880209.2021.1973038 PMC8451693

[46] Shao C, Yang F, Miao S, Liu W, Wang C, Shu Y, et al. Role of hypoxia-induced exosomes in tumor biology. Mol Cancer. 2018;17(1):120. 30098600 10.1186/s12943-018-0869-y PMC6087002

[47] Roma-Rodrigues C, Mendes R, Baptista PV, Fernandes AR. Targeting tumor microenvironment for cancer therapy. Int J Mol Sci. 2019;20(4):840. 30781344 10.3390/ijms20040840 PMC6413095

[48] Jing X, Yang F, Shao C, Wei K, Xie M, Shen H, et al. Role of hypoxia in cancer therapy by regulating the tumor microenvironment. Mol Cancer. 2019;18(1):157. 31711497 10.1186/s12943-019-1089-9 PMC6844052

[49] Huang Y, Lin D, Taniguchi CM. Hypoxia inducible factor (HIF) in the tumor microenvironment: friend or foe? Sci China Life Sci. 2017;60(10):1114–24. 29039125 10.1007/s11427-017-9178-y PMC6131113

[50] Liang Y, Zheng T, Song R, Wang J, Yin D, Wang L, et al. Hypoxia-mediated sorafenib resistance can be overcome by EF24 through Von Hippel-Lindau tumor suppressor-dependent HIF-1alpha inhibition in hepatocellular carcinoma. Hepatology. 2013;57(5):1847–57. 23299930 10.1002/hep.26224

[51] Shi J, Li J, Li J, Li R, Wu X, Gao F, et al. Synergistic breast cancer suppression efficacy of doxorubicin by combination with glycyrrhetinic acid as an angiogenesis inhibitor. Phytomedicine. 2021;81: 153408. 33234363 10.1016/j.phymed.2020.153408

[52] Li J, Wu Y, Wang D, Zou L, Fu C, Zhang J, et al. Oridonin synergistically enhances the anti-tumor efficacy of doxorubicin against aggressive breast cancer via pro-apoptotic and anti-angiogenic effects. Pharmacol Res. 2019;146: 104313. 31202781 10.1016/j.phrs.2019.104313

[53] Kong F, Zhao L, Wang N, Zhang D, Wang Z, Mei Q, et al. The FJQR has synergistic effect with fluoropyrimidine in the maintenance treatment for HER-2 negative gastric cancer. Recent Pat Anticancer Drug Discov. 2024;19(2):165–75. 38214356 10.2174/1574892818666230522161742 PMC10909912

[54] Han L, Zhang W, Li X, He Q, Han J, Zhang Y, et al. Investigating the anti-angiogenic effects of Fufang Kushen Injection in combination with cisplatin using a zebrafish model. Pak J Pharm Sci. 2020;33(5):1955–60. 33824101

[55] Wang D, Xu Y, Huang T, Peng W, Zhu D, Zhou X, et al. Clinical efficacy and safety of NSCLC ancillary treatment with compound Kushen injection through immunocompetence regulation: a systematic review and meta-analysis. Phytomedicine. 2022;104: 154315. 35868145 10.1016/j.phymed.2022.154315

[56] Engel JL, Zhang X, Wu M, Wang Y, Espejo Valle-Inclan J, Hu Q, et al. The Fanconi anemia pathway induces chromothripsis and ecDNA-driven cancer drug resistance. Cell. 2024;187(21):6055-70.e22. 39181133 10.1016/j.cell.2024.08.001 PMC11490392

[57] Kaljunen H, Taavitsainen S, Kaarijarvi R, Takala E, Paakinaho V, Nykter M, et al. Fanconi anemia pathway regulation by FANCI in prostate cancer. Front Oncol. 2023;13:1260826. 38023254 10.3389/fonc.2023.1260826 PMC10643534

[58] Fan XZ, Chen YF, Zhang SB, He DH, Wei SF, Wang Q, et al. Centipeda minima extract sensitizes lung cancer cells to DNA-crosslinking agents via targeting Fanconi anemia pathway. Phytomedicine. 2021;91: 153689. 34446320 10.1016/j.phymed.2021.153689

[59] Fan Z, Duan J, Wang L, Xiao S, Li L, Yan X, et al. PTK2 promotes cancer stem cell traits in hepatocellular carcinoma by activating Wnt/beta-catenin signaling. Cancer Lett. 2019;450:132–43. 30849480 10.1016/j.canlet.2019.02.040

[60] Shibue T, Weinberg RA. EMT, CSCs, and drug resistance: the mechanistic link and clinical implications. Nat Rev Clin Oncol. 2017;14(10):611–29. 28397828 10.1038/nrclinonc.2017.44 PMC5720366

[61] Tao H, Ding X, Wu J, Liu S, Sun W, Nie M, et al. Beta-asarone increases chemosensitivity by inhibiting tumor glycolysis in gastric cancer. Evid Based Complement Alternat Med. 2020;2020:6981520. 32351601 10.1155/2020/6981520 PMC7171649

[62] Bai B, Ma Y, Liu D, Zhang Y, Zhang W, Shi R, et al. DNA damage caused by chemotherapy has duality, and traditional Chinese medicine may be a better choice to reduce its toxicity. Front Pharmacol. 2024;15:1483160. 39502534 10.3389/fphar.2024.1483160 PMC11534686

[63] Katsuya H, Tamura K. Side effects of chemotherapy. Nihon Rinsho. 2015;73(Suppl 2):39–44. 25831721

[64] Li B, Shao H, Gao L, Li H, Sheng H, Zhu L. Nano-drug co-delivery system of natural active ingredients and chemotherapy drugs for cancer treatment: a review. Drug Deliv. 2022;29(1):2130–61. 35815678 10.1080/10717544.2022.2094498 PMC9275501

[65] Chen Y, Cheng CS, Tan HY, Tam CW, Wang N, Feng Y. Efficacy of herbal medicines intervention for colorectal cancer patients with chemotherapy-induced gastrointestinal toxicity - a systematic review and meta-analysis. Front Oncol. 2021;11: 629132. 33869014 10.3389/fonc.2021.629132 PMC8044744

[66] Tan Y, Wang H, Xu B, Zhang X, Zhu G, Ge Y, et al. Chinese herbal medicine combined with oxaliplatin-based chemotherapy for advanced gastric cancer: a systematic review and meta-analysis of contributions of specific medicinal materials to tumor response. Front Pharmacol. 2022;13: 977708. 36091754 10.3389/fphar.2022.977708 PMC9453215

[67] Kruger K, Thomale J, Stojanovic N, Osmak M, Henninger C, Bormann S, et al. Platinum-induced kidney damage: Unraveling the DNA damage response (DDR) of renal tubular epithelial and glomerular endothelial cells following platinum injury. Biochim Biophys Acta. 2015;1853(3):685–98. 25565603 10.1016/j.bbamcr.2014.12.033

[68] Samodelov SL, Gai Z, Kullak-Ublick GA, Visentin M. Renal reabsorption of folates: pharmacological and toxicological snapshots. Nutrients. 2019;11(10):2353. 31581752 10.3390/nu11102353 PMC6836044

[69] Lefebvre J, Glezerman IG. Kidney toxicities associated with novel cancer therapies. Adv Chronic Kidney Dis. 2017;24(4):233–40. 28778363 10.1053/j.ackd.2017.05.006

[70] Cai Y, Huang C, Zhou M, Xu S, Xie Y, Gao S, et al. Role of curcumin in the treatment of acute kidney injury: research challenges and opportunities. Phytomedicine. 2022;104: 154306. 35809376 10.1016/j.phymed.2022.154306

[71] Thongnuanjan P, Soodvilai S, Fongsupa S, Thipboonchoo N, Chabang N, Munyoo B, et al. Panduratin A derivative protects against cisplatin-induced apoptosis of renal proximal tubular cells and kidney injury in mice. Molecules. 2021;26(21):6642. 34771049 10.3390/molecules26216642 PMC8588142

[72] Yang Z, Ning R, Liu Q, Zang R, Liu S, Sun S. Umbelliferone attenuates cisplatin-induced acute kidney injury by inhibiting oxidative stress and inflammation via NRF2. Physiol Rep. 2023;11(23): e15879. 38030388 10.14814/phy2.15879 PMC10686806

[73] Wang XW, Tian RM, Yang YQ, Wang K, Li EN, Han XD, et al. Tripterygium glycoside fraction n2 ameliorates adriamycin-induced nephrotic syndrome in rats by suppressing apoptosis. J Ethnopharmacol. 2020;257: 112789. 32234597 10.1016/j.jep.2020.112789

[74] Qi L, Luo Q, Zhang Y, Jia F, Zhao Y, Wang F. Advances in toxicological research of the anticancer drug cisplatin. Chem Res Toxicol. 2019;32(8):1469–86. 31353895 10.1021/acs.chemrestox.9b00204

[75] Chen C, Ai QD, Wei YH. Kanglaite enhances the efficacy of cisplatin in suppression of hepatocellular carcinoma via inhibiting CKLF1 mediated NF-kappaB pathway and regulating transporter mediated drug efflux. J Ethnopharmacol. 2021;264: 113388. 32918990 10.1016/j.jep.2020.113388

[76] Huang X, Wang J, Lin W, Zhang N, Du J, Long Z, et al. Kanglaite injection plus platinum-based chemotherapy for stage III/IV non-small cell lung cancer: a meta-analysis of 27 RCTs. Phytomedicine. 2020;67: 153154. 31926475 10.1016/j.phymed.2019.153154

[77] Gazwi HSS, Zaki AH, Abd Allah NAR, Gomaa AT, Milosevic M, Al-Rejaie SS, et al. Mitigation of cisplatin-induced hepatotoxicity by Salvia officinalis: attenuation of oxidative damage and inflammation in rats. Free Radic Biol Med. 2024;222:62–71. 38852878 10.1016/j.freeradbiomed.2024.06.005

[78] Eisa NH, El-Sherbiny M, Abo El-Magd NF. Betulin alleviates cisplatin-induced hepatic injury in rats: Targeting apoptosis and Nek7-independent NLRP3 inflammasome pathways. Int Immunopharmacol. 2021;99: 107925. 34217992 10.1016/j.intimp.2021.107925

[79] Zhu L, Du J, Dai Y, Shen Y, Li H, Zhang Q, et al. Morinda officinalis iridoid glycosides alleviate methotrexate-induced liver injury in CIA rats by increasing liver autophagy and improving lipid metabolism homeostasis. J Ethnopharmacol. 2024;333: 118486. 38914148 10.1016/j.jep.2024.118486

[80] McGowan JV, Chung R, Maulik A, Piotrowska I, Walker JM, Yellon DM. Anthracycline chemotherapy and cardiotoxicity. Cardiovasc Drugs Ther. 2017;31(1):63–75. 28185035 10.1007/s10557-016-6711-0 PMC5346598

[81] Zhang X, Lv S, Zhang W, Jia Q, Wang L, Ding Y, et al. Shenmai injection improves doxorubicin cardiotoxicity via miR-30a/Beclin 1. Biomed Pharmacother. 2021;139: 111582. 33895525 10.1016/j.biopha.2021.111582

[82] Li W, Zhang Y, Wang X, Cao J, Qian W, Ling G, et al. Qishen granule protects against doxorubicin-induced cardiotoxicity by coordinating MDM2-p53-mediated mitophagy and mitochondrial biogenesis. Oxid Med Cell Longev. 2022;2022:4344677. 36120600 10.1155/2022/4344677 PMC9473341

[83] Chen L, Qin Z, Ruan ZB. Hyperoside alleviates doxorubicin-induced myocardial cells apoptosis by inhibiting the apoptosis signal-regulating kinase 1/p38 pathway. PeerJ. 2023;11: e15315. 37220525 10.7717/peerj.15315 PMC10200097

[84] Wei S, Ma W, Jiang C, Liu J, Liu J, Zhang B, et al. Hyperoside prevents doxorubicin-induced cardiotoxicity by inhibiting NOXs/ROS/NLRP3 inflammasome signaling pathway. Phytother Res. 2023;37(9):4196–209. 37246409 10.1002/ptr.7900

[85] Cheng Y, Wu X, Nie X, Wu Y, Zhang C, Lee SM, et al. Natural compound glycyrrhetinic acid protects against doxorubicin-induced cardiotoxicity by activating the Nrf2/HO-1 signaling pathway. Phytomedicine. 2022;106: 154407. 36070662 10.1016/j.phymed.2022.154407

[86] Loprinzi CL, Lacchetti C, Bleeker J, Cavaletti G, Chauhan C, Hertz DL, et al. Prevention and management of chemotherapy-induced peripheral neuropathy in survivors of adult cancers: ASCO guideline update. J Clin Oncol. 2020;38(28):3325–48. 32663120 10.1200/JCO.20.01399

[87] Seretny M, Currie GL, Sena ES, Ramnarine S, Grant R, MacLeod MR, et al. Incidence, prevalence, and predictors of chemotherapy-induced peripheral neuropathy: a systematic review and meta-analysis. Pain. 2014;155(12):2461–70. 25261162 10.1016/j.pain.2014.09.020

[88] Jongen JL, Broijl A, Sonneveld P. Chemotherapy-induced peripheral neuropathies in hematological malignancies. J Neurooncol. 2015;121(2):229–37. 25326770 10.1007/s11060-014-1632-x

[89] da Costa R, Passos GF, Quintao NLM, Fernandes ES, Maia J, Campos MM, et al. Taxane-induced neurotoxicity: pathophysiology and therapeutic perspectives. Br J Pharmacol. 2020;177(14):3127–46. 32352155 10.1111/bph.15086 PMC7312267

[90] Han J, Lai H, Li W, Liao H, Xiao C, Li X, et al. Efficacy and safety of traditional plant-based medicines for preventing chronic oxaliplatin-induced peripheral neurotoxicity in patients with colorectal cancer: a systematic review and meta-analysis with core herb contribution. J Ethnopharmacol. 2024;326: 117735. 38211824 10.1016/j.jep.2024.117735

[91] Matsumura Y, Futagami M, Baba T, Soeda S, Watari H, Terada Y, et al. Effectiveness of the traditional japanese medicine goshajinkigan in preventing paclitaxel-induced peripheral neuropathy: a multicenter randomized comparative trial. Integr Cancer Ther. 2024;23:15347354241278636. 39256982 10.1177/15347354241278635 PMC11403691

[92] Higuchi H, Yamamoto S, Ushio S, Kawashiri T, Egashira N. Goshajinkigan reduces bortezomib-induced mechanical allodynia in rats: Possible involvement of kappa opioid receptor. J Pharmacol Sci. 2015;129(3):196–9. 26598003 10.1016/j.jphs.2015.09.004

[93] Howard GR, Jost TA, Yankeelov TE, Brock A. Quantification of long-term doxorubicin response dynamics in breast cancer cell lines to direct treatment schedules. PLoS Comput Biol. 2022;18(3): e1009104. 35358172 10.1371/journal.pcbi.1009104 PMC9004764

[94] Nicolussi S, Drewe J, Butterweck V, Schwabedissen HEMZ. Clinical relevance of St. John’s wort drug interactions revisited. Br J Pharmacol. 2020;177(6):1212–26. 31742659 10.1111/bph.14936 PMC7056460

[95] Boyle DA. The perils of St. John’s wort in cancer care: Nursing considerations. Nursing. 2024;54(11):35–9. 39497667 10.1097/NSG.0000000000000078

[96] Bilgi N, Bell K, Ananthakrishnan AN, Atallah E. Imatinib and Panax ginseng: a potential interaction resulting in liver toxicity. Ann Pharmacother. 2010;44(5):926–8. 20332334 10.1345/aph.1M715

[97] Bossaer JB, Odle BL. Probable etoposide interaction with Echinacea. J Diet Suppl. 2012;9(2):90–5. 22607644 10.3109/19390211.2012.682643

[98] Dudas B, Bagdad Y, Picard M, Perahia D, Miteva MA. Machine learning and structure-based modeling for the prediction of UDP-glucuronosyltransferase inhibition. iScience. 2022;25(11):105290. 36304105 10.1016/j.isci.2022.105290 PMC9593791

[99] Liu D, Zhang L, Duan LX, Wu JJ, Hu M, Liu ZQ, et al. Potential of herb-drug/herb interactions between substrates and inhibitors of UGTs derived from herbal medicines. Pharmacol Res. 2019;150: 104510. 31678209 10.1016/j.phrs.2019.104510

[100] Xiao L, Zhu L, Li W, Li C, Cao Y, Ge G, et al. New insights into SN-38 glucuronidation: evidence for the important role of UDP glucuronosyltransferase 1A9. Basic Clin Pharmacol Toxicol. 2018;122(4):424–8. 29076612 10.1111/bcpt.12929

[101] Hirose K, Yamashita K, Takada H, Kaneda N, Fukami K, Maruo E, et al. Usefulness of one-point plasma SN-38G/SN-38 concentration ratios as a substitute for UGT1A1 genetic information after irinotecan administration. Int J Clin Oncol. 2014;19(2):397–402. 23605141 10.1007/s10147-013-0558-1

[102] Satoh T, Igarashi A, Tanno M, Yamada K, Takahashi-Suzuki N, Watanabe K. Inhibitory effects of baicalein derived from japanese traditional herbal medicine on SN-38 glucuronidation. J Pharm Pharm Sci. 2018;21(1):195–206. 29891024 10.18433/jpps29783

[103] Zhang XS, Zhao ZQ, Qin ZS, Wu K, Xia TF, Pang LQ. Herb-drug interaction between irinotecan and psoralidin-containing herbs. Eur J Drug Metab Pharmacokinet. 2015;40(4):481–4. 25216634 10.1007/s13318-014-0223-8

[104] Meza-Junco J, Chu QS, Christensen O, Rajagopalan P, Das S, Stefanyschyn R, et al. UGT1A1 polymorphism and hyperbilirubinemia in a patient who received sorafenib. Cancer Chemother Pharmacol. 2009;65(1):1–4. 19672597 10.1007/s00280-009-1096-4

[105] Hu ZP, Yang XX, Chen X, Cao J, Chan E, Duan W, et al. A mechanistic study on altered pharmacokinetics of irinotecan by St. John’s wort Curr Drug Metab. 2007;8(2):157–71. 17305494 10.2174/138920007779815995

[106] Hu Z, Yang X, Ho PC, Chan E, Chan SY, Xu C, et al. St. John’s Wort modulates the toxicities and pharmacokinetics of CPT-11 (irinotecan) in rats. Pharm Res. 2005;22(6):902–14. 15948034 10.1007/s11095-005-4585-0

[107] Hu ZP, Yang XX, Chan SY, Xu AL, Duan W, Zhu YZ, et al. St. John’s wort attenuates irinotecan-induced diarrhea via down-regulation of intestinal pro-inflammatory cytokines and inhibition of intestinal epithelial apoptosis. Toxicol Appl Pharmacol. 2006;216(2):225–37. 17015070 10.1016/j.taap.2006.05.020

[108] Wang B, Shen C, Li Y, Zhang T, Huang H, Ren J, et al. Oridonin overcomes the gemcitabine resistant PANC-1/Gem cells by regulating GST pi and LRP/1 ERK/JNK signalling. Onco Targets Ther. 2019;12:5751–65. 31410021 10.2147/OTT.S208924 PMC6645696

[109] Tan H, Wang F, Hu J, Duan X, Bai W, Wang X, et al. Inhibitory interaction of flavonoids with organic cation transporter 2 and their structure-activity relationships for predicting nephroprotective effects. J Appl Toxicol. 2023;43(10):1421–35. 37057715 10.1002/jat.4474

[110] Wang G, Bi Y, Xiong H, Bo T, Han L, Zhou L, et al. Wedelolactone protects against cisplatin-induced nephrotoxicity in mice via inhibition of organic cation transporter 2. Hum Exp Toxicol. 2021;40(12):S447–59. 34592875 10.1177/09603271211047915

[111] Wang FH, Tan HX, Hu JH, Duan XY, Bai WT, Wang XB, et al. Inhibitory interaction of flavonoids with organic anion transporter 3 and their structure-activity relationships for predicting nephroprotective effects. J Asian Nat Prod Res. 2024;26(3):353–71. 37589480 10.1080/10286020.2023.2240722

[112] Fan X, Bai J, Hu M, Xu Y, Zhao S, Sun Y, et al. Drug interaction study of flavonoids toward OATP1B1 and their 3D structure activity relationship analysis for predicting hepatoprotective effects. Toxicology. 2020;437: 152445. 32259555 10.1016/j.tox.2020.152445

[113] Boonnop R, Meetam P, Siangjong L, Tuchinda P, Thongphasuk P, Soodvilai S, et al. Black ginger extract and its active compound, 5,7-dimethoxyflavone, increase intestinal drug absorption via efflux drug transporter inhibitions. Drug Metab Pharmacokinet. 2023;50: 100500. 36948091 10.1016/j.dmpk.2023.100500

[114] Bae JK, Kim YJ, Chae HS, Kim DY, Choi HS, Chin YW, et al. Korean red ginseng extract enhances paclitaxel distribution to mammary tumors and its oral bioavailability by P-glycoprotein inhibition. Xenobiotica. 2017;47(5):450–9. 27189791 10.1080/00498254.2016.1182233

[115] Strope JD, Peer CJ, Sissung TM, Hall OM, Huang PA, Harris EM, et al. Botryllamide G is an ABCG2 inhibitor that improves lapatinib delivery in mouse brain. Cancer Biol Ther. 2020;21(3):223–30. 31709896 10.1080/15384047.2019.1683324 PMC7012088

[116] Rouibah H, Kebsa W, Lahouel M, Zihlif M, Ahram M, Aburmaileh B, et al. Algerian propolis: between protection of normal cells and potentialisation of the anticancer effects of doxorubicin against breast cancer cells via P-glycoprotein inhibition and cell cycle arrest in the S phase. J Physiol Pharmacol. 2021. 10.26402/jpp.2021.2.09. 34374660 10.26402/jpp.2021.2.09

[117] Elkady WM, Ayoub IM, Abdel-Mottaleb Y, ElShafie MF, Wink M. *Euryops pectinatus* L. flower extract inhibits P-glycoprotein and reverses multi-drug resistance in cancer cells: a mechanistic study. Molecules. 2020;25(3):647. 32028621 10.3390/molecules25030647 PMC7038149

[118] Abd-Ellatef GEF, Gazzano E, El-Desoky AH, Hamed AR, Kopecka J, Belisario DC, et al. Glabratephrin reverses doxorubicin resistance in triple negative breast cancer by inhibiting P-glycoprotein. Pharmacol Res. 2022;175: 105975. 34785319 10.1016/j.phrs.2021.105975

[119] Sun W, Wong ILK, Law HK, Su X, Chan TCF, Sun G, et al. In vivo reversal of P-glycoprotein-mediated drug resistance in a breast cancer xenograft and in leukemia models using a novel, potent, and nontoxic epicatechin EC31. Int J Mol Sci. 2023;24(5):4377. 36901808 10.3390/ijms24054377 PMC10002220

[120] Ben Toumia I, Sobeh M, Ponassi M, Banelli B, Dameriha A, Wink M, et al. A methanol extract of scabiosa atropurpurea enhances doxorubicin cytotoxicity against resistant colorectal cancer cells in vitro. Molecules. 2020;25(22):5265. 33198146 10.3390/molecules25225265 PMC7697796

[121] Pellegrini E, Multari G, Gallo FR, Vecchiotti D, Zazzeroni F, Condello M, et al. A natural product, voacamine, sensitizes paclitaxel-resistant human ovarian cancer cells. Toxicol Appl Pharmacol. 2022;434: 115816. 34856211 10.1016/j.taap.2021.115816

[122] Yang L, Zhang C, Chen J, Zhang S, Pan G, Xin Y, et al. Shenmai injection suppresses multidrug resistance in MCF-7/ADR cells through the MAPK/NF-kappaB signalling pathway. Pharm Biol. 2020;58(1):276–85. 32251615 10.1080/13880209.2020.1742167 PMC7170370

[123] Liao D, Zhang W, Gupta P, Lei ZN, Wang JQ, Cai CY, et al. Tetrandrine interaction with ABCB1 reverses multidrug resistance in cancer cells through competition with anti-cancer drugs followed by downregulation of ABCB1 expression. Molecules. 2019;24(23):4383. 31801248 10.3390/molecules24234383 PMC6930469

[124] Soleymani S, Bahramsoltani R, Rahimi R, Abdollahi M. Clinical risks of St John’s Wort (*Hypericum perforatum*) co-administration. Expert Opin Drug Metab Toxicol. 2017;13(10):1047–62. 28885074 10.1080/17425255.2017.1378342

[125] El Biali M, Wolfl-Duchek M, Jackwerth M, Mairinger S, Weber M, Bamminger K, et al. St. John’s wort extract with a high hyperforin content does not induce P-glycoprotein activity at the human blood-brain barrier. Clin Transl Sci. 2024;17(5):e13804. 38700454 10.1111/cts.13804 PMC11067874

[126] Chan WJ, Adiwidjaja J, McLachlan AJ, Boddy AV, Harnett JE. Interactions between natural products and cancer treatments: underlying mechanisms and clinical importance. Cancer Chemother Pharmacol. 2023;91(2):103–19. 36707434 10.1007/s00280-023-04504-z PMC9905199

[127] Zhou Q, Ye Z, Ruan Z, Zeng S. Investigation on modulation of human P-gp by multiple doses of Radix Astragali extract granules using fexofenadine as a phenotyping probe. J Ethnopharmacol. 2013;146(3):744–9. 23422332 10.1016/j.jep.2013.01.037

[128] Cox MC, Low J, Lee J, Walshe J, Denduluri N, Berman A, et al. Influence of garlic (*Allium sativum*) on the pharmacokinetics of docetaxel. Clin Cancer Res. 2006;12(15):4636–40. 16899612 10.1158/1078-0432.CCR-06-0388

[129] Hwang SW, Han HS, Lim KY, Han JY. Drug interaction between complementary herbal medicines and gefitinib. J Thorac Oncol. 2008;3(8):942–3. 18670318 10.1097/JTO.0b013e3181803f1e

[130] Thomas-Schoemann A, Blanchet B, Bardin C, Noe G, Boudou-Rouquette P, Vidal M, et al. Drug interactions with solid tumour-targeted therapies. Crit Rev Oncol Hematol. 2014;89(1):179–96. 24041628 10.1016/j.critrevonc.2013.08.007

[131] Harkey MR, Henderson GL, Gershwin ME, Stern JS, Hackman RM. Variability in commercial ginseng products: an analysis of 25 preparations. Am J Clin Nutr. 2001;73(6):1101–6. 11382666 10.1093/ajcn/73.6.1101

[132] Benkovic V, Horvat Knezevic A, Brozovic G, Knezevic F, Dikic D, Bevanda M, et al. Enhanced antitumor activity of irinotecan combined with propolis and its polyphenolic compounds on Ehrlich ascites tumor in mice. Biomed Pharmacother. 2007;61(5):292–7. 17412551 10.1016/j.biopha.2007.02.012

[133] Lisicic D, Benkovic V, Ethikic D, Blazevic AS, Mihaljevic J, Orsolic N, et al. Addition of propolis to irinotecan therapy prolongs survival in ehrlich ascites tumor-bearing mice. Cancer Biother Radiopharm. 2014;29(2):62–9. 24383762 10.1089/cbr.2013.1535 PMC3929170

[134] Zhang YW, Bao MH, Hu L, Qu Q, Zhou HH. Dose-response of oridonin on hepatic cytochromes P450 mRNA expression and activities in mice. J Ethnopharmacol. 2014;155(1):714–20. 24933226 10.1016/j.jep.2014.06.009

[135] Kazantseva L, Becerra J, Santos-Ruiz L. Oridonin enhances antitumor effects of doxorubicin in human osteosarcoma cells. Pharmacol Rep. 2022;74(1):248–56. 34427908 10.1007/s43440-021-00324-1 PMC8786785

[136] Cole S, Kerwash E, Andersson A. A summary of the current drug interaction guidance from the European Medicines Agency and considerations of future updates. Drug Metab Pharmacokinet. 2020;35(1):2–11. 31996310 10.1016/j.dmpk.2019.11.005

[137] Zhao P, Zhang L, Grillo JA, Liu Q, Bullock JM, Moon YJ, et al. Applications of physiologically based pharmacokinetic (PBPK) modeling and simulation during regulatory review. Clin Pharmacol Ther. 2011;89(2):259–67. 21191381 10.1038/clpt.2010.298

[138] Brantley SJ, Argikar AA, Lin YS, Nagar S, Paine MF. Herb-drug interactions: challenges and opportunities for improved predictions. Drug Metab Dispos. 2014;42(3):301–17. 24335390 10.1124/dmd.113.055236 PMC3935140

[139] Pilla Reddy V, Jo H, Neuhoff S. Food constituent- and herb-drug interactions in oncology: influence of quantitative modelling on drug labelling. Br J Clin Pharmacol. 2021;87(10):3988–4000. 33733472 10.1111/bcp.14822

[140] Adiwidjaja J, Boddy AV, McLachlan AJ. Physiologically based pharmacokinetic modelling of hyperforin to predict drug interactions with St John’s Wort. Clin Pharmacokinet. 2019;58(7):911–26. 30675694 10.1007/s40262-019-00736-6

[141] Gufford BT, Barr JT, Gonzalez-Perez V, Layton ME, White JR Jr, Oberlies NH, et al. Quantitative prediction and clinical evaluation of an unexplored herb-drug interaction mechanism in healthy volunteers. CPT Pharmacometrics Syst Pharmacol. 2015;4(12):701–10. 26904384 10.1002/psp4.12047 PMC4759704

[142] Fan J, Chen L, Lu X, Li M, Zhu L. The pharmacokinetic prediction of cyclosporin a after coadministration with Wuzhi capsule. AAPS PharmSciTech. 2019;20(6):247. 31286321 10.1208/s12249-019-1444-6

[143] Chen L, Ji N, Zhang M, Chen W. The influence of wuzhi capsule on the pharmacokinetics of cyclophosphamide. Recent Pat Anticancer Drug Discov. 2022;17(2):195–203. 34758719 10.2174/1574892816666211110152119

[144] He Q, Bu F, Wang Q, Li M, Lin J, Tang Z, et al. Examination of the impact of CYP3A4/5 on drug-drug interaction between schizandrol A/schizandrol B and tacrolimus (FK-506): a physiologically based pharmacokinetic modeling approach. Int J Mol Sci. 2022;23(9):4485. 35562875 10.3390/ijms23094485 PMC9103789

[145] Adiwidjaja J, Boddy AV, McLachlan AJ. Potential for pharmacokinetic interactions between Schisandra sphenanthera and bosutinib, but not imatinib: in vitro metabolism study combined with a physiologically-based pharmacokinetic modelling approach. Br J Clin Pharmacol. 2020;86(10):2080–94. 32250458 10.1111/bcp.14303 PMC7495297

[146] Vardell E. Natural medicines: a complementary and alternative medicines tool combining natural standard and the natural medicines comprehensive database. Med Ref Serv Q. 2015;34(4):461–70. 26496400 10.1080/02763869.2015.1082382

[147] Wu CS, Chen YH, Chen CL, Chien SK, Syifa N, Hung YC, et al. Constructing a bilingual website with validated database for Herb and Western medicine interactions using Ginseng, Ginkgo and Dong Quai as examples. BMC Complement Altern Med. 2019;19(1):335. 31775730 10.1186/s12906-019-2731-1 PMC6881993

[148] Birer-Williams C, Gufford BT, Chou E, Alilio M, VanAlstine S, Morley RE, et al. A new data repository for pharmacokinetic natural product-drug interactions: from chemical characterization to clinical studies. Drug Metab Dispos. 2020;48(10):1104–12. 32601103 10.1124/dmd.120.000054 PMC7543481

[149] DIDB® – The Drug Interaction Database: Certara; 2024. https://www.druginteractionsolutions.org/solutions/drug-interaction-database/.

[150] Zhang Y, Man Ip C, Lai YS, Zuo Z. Overview of current herb-drug interaction databases. Drug Metab Dispos. 2022;50(1):86–94. 34697080 10.1124/dmd.121.000420

[151] Hachad H, Ragueneau-Majlessi I, Levy RH. A useful tool for drug interaction evaluation: the University of Washington Metabolism and Transport Drug Interaction Database. Hum Genomics. 2010;5(1):61–72. 21106490 10.1186/1479-7364-5-1-61 PMC3500158

[152] UW Pharmacy’s Drug Interaction Database, built to promote medication safety, wins national innovation award: University of Washington; 2024. https://www.washington.edu/news/2022/01/13/uw-pharmacys-drug-interaction-database-built-to-promote-medication-safety-wins-national-innovation-award/.

[153] Rogala BG, Charpentier MM, Nguyen MK, Landolf KM, Hamad L, Gaertner KM. Oral anticancer therapy: management of drug interactions. J Oncol Pract. 2019;15(2):81–90. 30763198 10.1200/JOP.18.00483

[154] Hemaiswarya S, Prabhakar PK, Doble M. Computational methods to study herb-drug interactions. In: Hemaiswarya S, Prabhakar PK, Doble M, editors. Herb-drug combinations: a new complementary therapeutic strategy. Singapore: Springer Nature Singapore; 2022. p. 235–44.

[155] Zhai Y, Liu L, Zhang F, Chen X, Wang H, Zhou J, et al. Network pharmacology: a crucial approach in traditional Chinese medicine research. Chinese Medicine. 2025;20(1):8. 39800680 10.1186/s13020-024-01056-z PMC11725223

[156] Hashem S, Nisar S, Sageena G, Macha MA, Yadav SK, Krishnankutty R, et al. Therapeutic effects of curcumol in several diseases. An Overview Nutr Cancer. 2021;73(2):181–95. 32285707 10.1080/01635581.2020.1749676

[157] Xiying T, Ruxin GU, Jing T, Yu Z, RuiQian S, Gang Y, et al. Integrating network pharmacology and experimental validation to uncover the synergistic effects of Huangqi ()-Ezhu () with 5-fluorouracil in colorectal cancer models. J Tradit Chin Med. 2025;45(2):385–98. 40151125 10.19852/j.cnki.jtcm.2025.02.004 PMC11955770

[158] Das J, Somabattini RA, Chhabra N, Roy PP, Islam R, Dhaked DK, et al. Network pharmacology and bioinformatics based investigation of Phyllanthus fraternus: herb-drug interaction study. J Biomol Struct Dyn. 2025;43(3):1101–15. 38069602 10.1080/07391102.2023.2291167

[159] Zhai Y, Liu L, Zhang F, Chen X, Wang H, Zhou J, et al. Network pharmacology: a crucial approach in traditional Chinese medicine research. Chin Med. 2025;20(1):8. 39800680 10.1186/s13020-024-01056-z PMC11725223

[160] Xia F, Shukla M, Brettin T, Garcia-Cardona C, Cohn J, Allen JE, et al. Predicting tumor cell line response to drug pairs with deep learning. BMC Bioinformatics. 2018;19(Suppl 18):486. 30577754 10.1186/s12859-018-2509-3 PMC6302446

[161] Zagidullin B, Aldahdooh J, Zheng S, Wang W, Wang Y, Saad J, et al. DrugComb: an integrative cancer drug combination data portal. Nucleic Acids Res. 2019;47(W1):W43–51. 31066443 10.1093/nar/gkz337 PMC6602441

[162] Huang L, Li F, Sheng J, Xia X, Ma J, Zhan M, et al. DrugComboRanker: drug combination discovery based on target network analysis. Bioinformatics. 2014;30(12):i228–36. 24931988 10.1093/bioinformatics/btu278 PMC4058933

[163] Preuer K, Lewis RPI, Hochreiter S, Bender A, Bulusu KC, Klambauer G. DeepSynergy: predicting anti-cancer drug synergy with deep learning. Bioinformatics. 2018;34(9):1538–46. 29253077 10.1093/bioinformatics/btx806 PMC5925774

[164] Wang LL, Tafjord O, Cohan A, Jain S, Skjonsberg S, Schoenick C, et al. SUPP.AI: Finding Evidence for Supplement-Drug Interactions. 01 Sep 2019. arXiv:1909.08135. https://ui.adsabs.harvard.edu/abs/2019arXiv190908135W.

[165] SUPP.AI by AI2: The Allen Institute for Artificial Intelligence; 2024. https://supp.ai/.

[166] Noor F, Asif M, Ashfaq UA, Qasim M, Ul Qamar MT. Machine learning for synergistic network pharmacology: a comprehensive overview. Brief Bioinform. 2023;24(3):bbad120. 37031957 10.1093/bib/bbad120

[167] Zhang P, Zhang D, Zhou W, Wang L, Wang B, Zhang T, et al. Network pharmacology: towards the artificial intelligence-based precision traditional Chinese medicine. Brief Bioinform. 2023;25(1):bbad518. 38197310 10.1093/bib/bbad518 PMC10777171

[168] Spanakis M, Tzamali E, Tzedakis G, Koumpouzi C, Pediaditis M, Tsatsakis A, et al. Artificial intelligence models and tools for the assessment of drug-herb interactions. Pharmaceuticals (Basel). 2025;18(3):282. 40143062 10.3390/ph18030282 PMC11944892

[169] Chen HY, Chen JQ, Li JY, Huang HJ, Chen X, Zhang HY, et al. Deep learning and random forest approach for finding the optimal traditional chinese medicine formula for treatment of Alzheimer’s disease. J Chem Inf Model. 2019;59(4):1605–23. 30888812 10.1021/acs.jcim.9b00041

[170] Lei T, Li Y, Song Y, Li D, Sun H, Hou T. ADMET evaluation in drug discovery: 15. Accurate prediction of rat oral acute toxicity using relevance vector machine and consensus modeling. J Cheminform. 2016;8:6. 26839598 10.1186/s13321-016-0117-7 PMC4736633

[171] Altalib MK, Salim N. Similarity-based virtual screen using enhanced siamese deep learning methods. ACS Omega. 2022;7(6):4769–86. 35187297 10.1021/acsomega.1c04587 PMC8851658

[172] Luan X, Zhang LJ, Li XQ, Rahman K, Zhang H, Chen HZ, et al. Compound-based Chinese medicine formula: From discovery to compatibility mechanism. J Ethnopharmacol. 2020;254: 112687. 32105748 10.1016/j.jep.2020.112687

[173] Liu S, Qiao S, Wang S, Tao Z, Wang J, Tao J, et al. Intestinal bacteria are involved in Radix Glycyrrhizae and Radix Euphorbiae Pekinensis incompatibility. J Ethnopharmacol. 2021;273: 113839. 33476713 10.1016/j.jep.2021.113839

[174] Mariappan TT, Shen H, Marathe P. Endogenous biomarkers to assess drug-drug interactions by drug transporters and enzymes. Curr Drug Metab. 2017;18(8):757–68. 28738769 10.2174/1389200218666170724110818

[175] Poweleit EA, Vinks AA, Mizuno T. Artificial intelligence and machine learning approaches to facilitate therapeutic drug management and model-informed precision dosing. Ther Drug Monit. 2023;45(2):143–50. 36750470 10.1097/FTD.0000000000001078 PMC10378651

[176] Li S, Li X, Yang X, Lei Y, He M, Xiang X, et al. Corilagin enhances the anti-tumor activity of 5-FU by downregulating the expression of GRP 78. Sci Rep. 2023;13(1):22661. 38114593 10.1038/s41598-023-49604-1 PMC10730900

[177] Ham IH, Wang L, Lee D, Woo J, Kim TH, Jeong HY, et al. Curcumin inhibits the cancer-associated fibroblast-derived chemoresistance of gastric cancer through the suppression of the JAK/STAT3 signaling pathway. Int J Oncol. 2022;61(1):85. 35621145 10.3892/ijo.2022.5375 PMC9170354

[178] Bai Y, Xiong Y, Zhang YY, Cheng L, Liu H, Xu K, et al. Tangeretin synergizes with 5-fluorouracil to induce autophagy through microRNA-21 in colorectal cancer cells. Am J Chin Med. 2022;50(6):1681–701. 35848125 10.1142/S0192415X22500719

[179] Kshetrimayum V, Heisnam R, Keithellakpam OS, Radhakrishnanand P, Akula SJ, Mukherjee PK, et al. *Paris polyphylla* Sm. induces reactive oxygen species and caspase 3-mediated apoptosis in colorectal cancer cells in vitro and potentiates the therapeutic significance of fluorouracil and cisplatin. Plants (Basel). 2023;12(7):1446. 37050072 10.3390/plants12071446 PMC10097216

[180] Condello M, Vona R, Meschini S. *Prunus spinosa* extract sensitized hct116 spheroids to 5-fluorouracil toxicity, inhibiting autophagy. Int J Mol Sci. 2022;23(24):16098. 36555736 10.3390/ijms232416098 PMC9785163

[181] Liu H, Liu H, Zhou Z, Chung J, Zhang G, Chang J, et al. *Scutellaria baicalensis* enhances 5-fluorouracil-based chemotherapy via inhibition of proliferative signaling pathways. Cell Commun Signal. 2023;21(1):147. 37337282 10.1186/s12964-023-01156-7 PMC10278337

[182] Prompipak J, Senawong T, Sripa B, Ketterman AJ, Utaiwat S, Woranam K, et al. Anticancer effects of the combined Thai noni juice ethanolic extracts and 5-fluorouracil against cholangiocarcinoma cells in vitro and in vivo. Sci Rep. 2021;11(1):14866. 34290264 10.1038/s41598-021-94049-z PMC8295291

[183] Sun LT, Zhang LY, Shan FY, Shen MH, Ruan SM. Jiedu Sangen decoction inhibits chemoresistance to 5-fluorouracil of colorectal cancer cells by suppressing glycolysis via PI3K/AKT/HIF-1alpha signaling pathway. Chin J Nat Med. 2021;19(2):143–52. 33641785 10.1016/S1875-5364(21)60015-8

[184] Chen J, Liu J, Xu B, Cao Y, Liang X, Wu F, et al. Ethoxy-erianin phosphate and afatinib synergistically inhibit liver tumor growth and angiogenesis via regulating VEGF and EGFR signaling pathways. Toxicol Appl Pharmacol. 2022;438: 115911. 35143806 10.1016/j.taap.2022.115911

[185] Qi W, Yan X, Xu X, Song B, Sun L, Zhao D, et al. The effects of cytarabine combined with ginsenoside compound K synergistically induce DNA damage in acute myeloid leukemia cells. Biomed Pharmacother. 2020;132: 110812. 33059263 10.1016/j.biopha.2020.110812

[186] Han Q, Bai H, Xu Y, Zhou M, Zhou H, Dong X, et al. Solamargine induces autophagy-mediated apoptosis and enhances bortezomib activity in multiple myeloma. Clin Exp Pharmacol Physiol. 2022;49(6):674–85. 35294057 10.1111/1440-1681.13643 PMC9310729

[187] Bergel CC, Eryilmaz IE, Bulut E, Balaban RF, Egeli U, Cecener G. Synergistic anti-tumorigenic effects of cabazitaxel and usnic acid combination on metastatic castration-resistant prostate cancer cells. Anticancer Agents Med Chem. 2025;25(9):610–9. 39810522 10.2174/0118715206336754241015062614

[188] Wu H, Sun T, Bi R. Inhibition of insulin-like growth factor 1 signaling synergistically enhances the tumor suppressive role of triptolide in triple-negative breast cancer cells. Oncol Lett. 2019;18(1):822–9. 31289559 10.3892/ol.2019.10356 PMC6539977

[189] Chen JH, Lin TH, Chien YC, Chen CY, Lin CT, Kuo WW, et al. Aqueous extracts of *Ocimum gratissimum* sensitize hepatocellular carcinoma cells to cisplatin through BRCA1 inhibition. Int J Mol Sci. 2024;25(15):8424. 39125994 10.3390/ijms25158424 PMC11313253

[190] Sun Y, Chen Y, Xu M, Liu C, Shang H, Wang C. Shenmai injection supresses glycolysis and enhances cisplatin cytotoxicity in cisplatin-resistant A549/DDP cells via the AKT-mTOR-c-Myc signaling pathway. Biomed Res Int. 2020;2020:9243681. 32685545 10.1155/2020/9243681 PMC7327568

[191] Cai Z, Gao L, Hu K, Wang QM. Parthenolide enhances the metronomic chemotherapy effect of cyclophosphamide in lung cancer by inhibiting the NF-kB signaling pathway. World J Clin Oncol. 2024;15(7):895–907. 39071467 10.5306/wjco.v15.i7.895 PMC11271733

[192] Wei G, Sun J, Luan W, Hou Z, Wang S, Cui S, et al. Natural product albiziabioside A conjugated with pyruvate dehydrogenase kinase inhibitor dichloroacetate to induce apoptosis-ferroptosis-M2-TAMs polarization for combined cancer therapy. J Med Chem. 2019;62(19):8760–72. 31509699 10.1021/acs.jmedchem.9b00644

[193] Wei T, Xiaojun X, Peilong C. Magnoflorine improves sensitivity to doxorubicin (DOX) of breast cancer cells via inducing apoptosis and autophagy through AKT/mTOR and p38 signaling pathways. Biomed Pharmacother. 2020;121: 109139. 31707337 10.1016/j.biopha.2019.109139

[194] Liang L, Amin A, Cheung WY, Xu R, Yu R, Tang J, et al. Parameritannin A-2 from *Urceola huaitingii* enhances doxorubicin-induced mitochondria-dependent apoptosis by inhibiting the PI3K/Akt, ERK1/2 and p38 pathways in gastric cancer cells. Chem Biol Interact. 2020;316: 108924. 31843629 10.1016/j.cbi.2019.108924

[195] Lv LN, Wang XC, Tao LJ, Li HW, Li SY, Zheng FM. beta-Asarone increases doxorubicin sensitivity by suppressing NF-kappaB signaling and abolishes doxorubicin-induced enrichment of stem-like population by destabilizing Bmi1. Cancer Cell Int. 2019;19:153. 31171917 10.1186/s12935-019-0873-3 PMC6547485

[196] Frion-Herrera Y, Gabbia D, Diaz-Garcia A, Cuesta-Rubio O, Carrara M. Chemosensitizing activity of *Cuban propolis* and nemorosone in doxorubicin resistant human colon carcinoma cells. Fitoterapia. 2019;136: 104173. 31085307 10.1016/j.fitote.2019.104173

[197] Sanchez BG, Bort A, Mateos-Gomez PA, Rodriguez-Henche N, Diaz-Laviada I. Combination of the natural product capsaicin and docetaxel synergistically kills human prostate cancer cells through the metabolic regulator AMP-activated kinase. Cancer Cell Int. 2019;19:54. 30899201 10.1186/s12935-019-0769-2 PMC6408806

[198] Chen YY, Hua WX, Huang YH, Ding X. Polyphyllin VII enhances the sensitivity of prostate cancer cells to docetaxel by promoting mitochondrial dysfunction and inducing ferroptosis. Chem Biol Drug Des. 2025;105(2): e70053. 39871642 10.1111/cbdd.70053

[199] de Oliveira CE, Santiago KB, Conti BJ, Conte FL, Tasca KI, Romagnoli GG, et al. Brazilian green propolis: a novel tool to improve the cytotoxic and immunomodulatory action of docetaxel on MCF-7 breast cancer cells and on women monocyte. Phytother Res. 2022;36(1):448–61. 34862831 10.1002/ptr.7345

[200] Li H, Xu X, Zhang Y, Tang X, Li W. Tetrandrine enhances antitumor effects of the histone deacetylase inhibitor MS-275 in human cancer in a Bax- and p53-dependent manner. Eur J Pharmacol. 2020;888: 173575. 32950498 10.1016/j.ejphar.2020.173575

[201] Qin F, Wang CY, Kim D, Wang HS, Zhu YK, Lee SK, et al. Nitidumpeptins A and B, cyclohexapeptides isolated from *Zanthoxylum nitidum* var. tomentosum: structural elucidation, total synthesis, and antiproliferative activity in cancer cells. J Org Chem. 2021;86(2):1462–70. 33410687 10.1021/acs.joc.0c02057

[202] Jeong I, Song J, Bae SY, Lee SK. Overcoming the intrinsic gefitinib-resistance via downregulation of AXL in non-small Cell Lung cancer. J Cancer Prev. 2019;24(4):217–23. 31950021 10.15430/JCP.2019.24.4.217 PMC6951316

[203] Shen F, Ge C, Yuan P. Aloe-emodin induces autophagy and apoptotic cell death in non-small cell lung cancer cells via Akt/mTOR and MAPK signaling. Eur J Pharmacol. 2020;886: 173550. 32926915 10.1016/j.ejphar.2020.173550

[204] Yang H, Tong Z, Shen L, Sun YU, Hoffman RM, Huang J. *Brucea javanica* increases survival and enhances gemcitabine efficacy in a patient-derived orthotopic xenograft (PDOX) mouse model of pancreatic cancer. Anticancer Res. 2020;40(9):4969–78. 32878785 10.21873/anticanres.14500

[205] Lin X, Xu L, Gu M, Shao H, Yao L, Huang X. Gegen Qinlian Decoction reverses oxaliplatin resistance in colorectal cancer by inhibiting YTHDF1-regulated m6A modification of GLS1. Phytomedicine. 2024;133: 155906. 39089089 10.1016/j.phymed.2024.155906

[206] Kim BR, Jeong YA, Jo MJ, Park SH, Na YJ, Kim JL, et al. Genipin enhances the therapeutic effects of oxaliplatin by upregulating BIM in colorectal cancer. Mol Cancer Ther. 2019;18(4):751–61. 30787174 10.1158/1535-7163.MCT-18-0196

[207] Liu Y, Shi C, He Z, Zhu F, Wang M, He R, et al. Inhibition of PI3K/AKT signaling via ROS regulation is involved in Rhein-induced apoptosis and enhancement of oxaliplatin sensitivity in pancreatic cancer cells. Int J Biol Sci. 2021;17(2):589–602. 33613115 10.7150/ijbs.49514 PMC7893580

[208] El-Hanboshy SM, Helmy MW, Abd-Alhaseeb MM. Catalpol synergistically potentiates the anti-tumour effects of regorafenib against hepatocellular carcinoma via dual inhibition of PI3K/Akt/mTOR/NF-kappaB and VEGF/VEGFR2 signaling pathways. Mol Biol Rep. 2021;48(11):7233–42. 34596810 10.1007/s11033-021-06715-0

[209] Zhu XF, Sun ZL, Ma J, Hu B, Yu MC, Liu XJ, et al. Synergistic anticancer effect of flavonoids from Sophora alopecuroides with Sorafenib against hepatocellular carcinoma. Phytother Res. 2023;37(2):592–610. 36180975 10.1002/ptr.7637

[210] Fan K, Huang H, Zhao Y, Xie T, Zhu ZY, Xie ML. Osthole increases the sensitivity of liver cancer to sorafenib by inhibiting cholesterol metabolism. Nutr Cancer. 2022;74(10):3640–50. 35706361 10.1080/01635581.2022.2087885

[211] Ho WY, Liew SS, Yeap SK, Alitheen NB. Synergistic cytotoxicity between elephantopus scaber and tamoxifen on MCF-7-derived multicellular tumor spheroid. Evid Based Complement Alternat Med. 2021;2021:6355236. 34712346 10.1155/2021/6355236 PMC8548115

[212] Giakoumettis D, Pourzitaki C, Vavilis T, Tsingotjidou A, Kyriakoudi A, Tsimidou M, et al. *Crocus sativus* L. causes a non apoptotic calpain dependent death in C6 rat glioma cells, exhibiting a synergistic effect with temozolomide. Nutr Cancer. 2019;71(3):491–507. 30273051 10.1080/01635581.2018.1506493

[213] Lozon L, Saleh E, Menon V, Ramadan WS, Amin A, El-Awady R. Effect of safranal on the response of cancer cells to topoisomerase I inhibitors: does sequence matter? Front Pharmacol. 2022;13: 938471. 36120345 10.3389/fphar.2022.938471 PMC9479137

[214] Luo PQ, Zhang LX, Chen ZM, Wang G, Zhu H, Ying S, et al. Effects and mechanisms of trifluridine alone or in combination with cryptotanshinone in inhibiting malignant biological behavior of gastric cancer. Cell Cycle. 2023;22(12):1463–77. 37272203 10.1080/15384101.2023.2215678 PMC10281482

[215] Zhang JT, Liu P, Wang WL, Xie XX, He TH, Cui YR, et al. Bletilla striata polysaccharide improves toxic and side effects induced by 5-FU: an untargeted metabolomics study. Zhongguo Zhong Yao Za Zhi. 2023;48(13):3612–22. 37474994 10.19540/j.cnki.cjcmm.20230413.705

[216] Hong M, Chen D, Hong Z, Tang K, Yao Y, Chen L, et al. Ex vivo and in vivo chemoprotective activity and potential mechanism of Martynoside against 5-fluorouracil-induced bone marrow cytotoxicity. Biomed Pharmacother. 2021;138: 111501. 33765584 10.1016/j.biopha.2021.111501

[217] Yu L, Qin JY, Sun C, Peng F, Chen Y, Wang SJ, et al. Xianglian Pill combined with 5-fluorouracil enhances antitumor activity and reduces gastrointestinal toxicity in gastric cancer by regulating the p38 MAPK/NF-kappaB signaling pathway. J Ethnopharmacol. 2024;326: 117988. 38428657 10.1016/j.jep.2024.117988

[218] Teng H, Sun X, Eglitis R, Wang X, Zhang W, Wang H, et al. Chiisanoside from the leaves of acanthopanax sessiliflorus can resist cisplatin-induced ototoxicity by maintaining cytoskeletal homeostasis and inhibiting ferroptosis. J Agric Food Chem. 2024;72(46):25720–42. 39505327 10.1021/acs.jafc.4c07994

[219] Di Y, Xu T, Tian Y, Ma T, Qu D, Wang Y, et al. Ursolic acid protects against cisplatin-induced ototoxicity by inhibiting oxidative stress and TRPV1-mediated Ca2+-signaling. Int J Mol Med. 2020;46(2):806–16. 32626955 10.3892/ijmm.2020.4633 PMC7307815

[220] Chen Y, Hu Z, Jiang J, Liu C, Gao S, Song M, et al. Evaluation of pharmacological and pharmacokinetic herb-drug interaction between irinotecan hydrochloride injection and Kangai injection in colorectal tumor-bearing mice and healthy rats. Front Pharmacol. 2023;14:1282062. 38094890 10.3389/fphar.2023.1282062 PMC10716275

[221] Dhanisha SS, Drishya S, Guruvayoorappan C. Pithecellobium dulce fruit extract mitigates cyclophosphamide-mediated toxicity by regulating proinflammatory cytokines. J Food Biochem. 2020;44(1): e13083. 31633209 10.1111/jfbc.13083

[222] Saetang J, Tedasen A, Sangkhathat S, Sangkaew N, Dokduang S, Prompat N, et al. The attenuation effect of low piperine Piper nigrum extract on doxorubicin-induced toxicity of blood chemical and immunological properties in mammary tumour rats. Pharm Biol. 2022;60(1):96–107. 34962450 10.1080/13880209.2021.2018470 PMC8735876

[223] Tajaldini M, Samadi F, Khosravi A, Ghasemnejad A, Asadi J. Protective and anticancer effects of orange peel extract and naringin in doxorubicin treated esophageal cancer stem cell xenograft tumor mouse model. Biomed Pharmacother. 2020;121: 109594. 31707344 10.1016/j.biopha.2019.109594

[224] Salem MM, Donia T, Abu-Khudir R, Ramadan H, Ali EMM, Mohamed TM. Propolis potentiates methotrexate anticancer mechanism and reduces its toxic effects. Nutr Cancer. 2020;72(3):460–80. 31318622 10.1080/01635581.2019.1640884

[225] Zhang MW, Sun X, Xu YW, Meng W, Tang Q, Gao H, et al. Curcumin relieves oxaliplatin-induced neuropathic pain via reducing inflammation and activating antioxidant response. Cell Biol Int. 2024;48(6):872–82. 38480956 10.1002/cbin.12153

[226] Sun L, Xu Y, Chen N, Zhang C, Wu A, Wang H, et al. Chinese herbal medicine (JianPi-BuShen) and completion rate of adjuvant chemotherapy for patients with stage II and III colon cancer: a randomized clinical trial. Eur J Cancer. 2024;213: 115109. 39509846 10.1016/j.ejca.2024.115109 PMC11622473

[227] Zhu XY, Guo DW, Lao QC, Xu YQ, Meng ZK, Xia B, et al. Sensitization and synergistic anti-cancer effects of Furanodiene identified in zebrafish models. Sci Rep. 2019;9(1):4541. 30872660 10.1038/s41598-019-40866-2 PMC6418268

[228] Feng M, Fan X, Shi J, Shan S, Li S, He S, et al. Terpenoids from quinoa reverse drug resistance of colon cancer by upregulating miR-495-3p. J Sci Food Agric. 2024;104(14):8916–27. 38962946 10.1002/jsfa.13718

[229] Fang SQ, Huang J, Zhang F, Ni HM, Chen QL, Zhu JR, et al. Pharmacokinetic interaction between a Chinese herbal formula Huosu Yangwei oral liquid and apatinib in vitro and in vivo. J Pharm Pharmacol. 2020;72(7):979–89. 32285478 10.1111/jphp.13268

[230] Lin KN, Jiang YL, Zhang SG, Huang SY, Li H. Grape seed proanthocyanidin extract reverses multidrug resistance in HL-60/ADR cells via inhibition of the PI3K/Akt signaling pathway. Biomed Pharmacother. 2020;125: 109885. 32007917 10.1016/j.biopha.2020.109885

[231] Qu X, Gao H, Zhai J, Sun J, Tao L, Zhang Y, et al. Astragaloside IV enhances cisplatin chemosensitivity in hepatocellular carcinoma by suppressing MRP2. Eur J Pharm Sci. 2020;148: 105325. 32259679 10.1016/j.ejps.2020.105325

[232] Kalthoff S, Paulusch S, Rupp A, Holdenrieder S, Hartmann G, Strassburg CP. The coffee ingredients caffeic acid and caffeic acid phenylethyl ester protect against irinotecan-induced leukopenia and oxidative stress response. Br J Pharmacol. 2020;177(18):4193–208. 32548889 10.1111/bph.15162 PMC7443465

[233] Man S, Li Y, Fan W, Gao W, Liu Z, Zhang Y, et al. Combination therapy of cyclophosphamide and Rhizoma Paridis Saponins on anti-hepatocarcinoma mice and effects on cytochrome p450 enzyme expression. Steroids. 2014;80:1–6. 24291418 10.1016/j.steroids.2013.11.015

[234] Shahid M, Ahmad A, Raish M, Bin Jardan YA, Alkharfy KM, Ahad A, et al. Herb-drug interaction: Effect of sinapic acid on the pharmacokinetics of dasatinib in rats. Saudi Pharm J. 2023;31(11): 101819. 37860687 10.1016/j.jsps.2023.101819 PMC10582055

[235] Luo F, Yang J, Yang X, Mi J, Ye T, Li G, et al. Saikosaponin D potentiates the antineoplastic effects of doxorubicin in drug-resistant breast cancer through perturbing NQO1-mediated intracellular redox balance. Phytomedicine. 2024;133: 155945. 39146878 10.1016/j.phymed.2024.155945

[236] Teng YN, Huang BH, Huang SY, Wu IT, Wu TS, Lee TE, et al. Cinnamophilin overcomes cancer multi-drug resistance via allosterically modulating human P-glycoprotein on both drug binding sites and ATPase binding sites. Biomed Pharmacother. 2021;144: 112379. 34794239 10.1016/j.biopha.2021.112379

[237] Wang T, Long F, Jiang G, Cai H, Jiang Q, Cheng K, et al. Pharmacokinetic properties of wogonin and its herb-drug interactions with docetaxel in rats with mammary tumors. Biomed Chromatogr. 2018;21:4264. 10.1002/bmc.4264 29679509

[238] Goey AK, Meijerman I, Rosing H, Marchetti S, Mergui-Roelvink M, Keessen M, et al. The effect of St. John’s wort on the pharmacokinetics of docetaxel. Clin Pharmacokinet. 2014;53(1):103–10. 24068654 10.1007/s40262-013-0102-5

[239] Han SY, Zhao HY, Zhou N, Zhou F, Li PP. Marsdenia tenacissima extract inhibits gefitinib metabolism in vitro by interfering with human hepatic CYP3A4 and CYP2D6 enzymes. J Ethnopharmacol. 2014;151(1):210–7. 24157377 10.1016/j.jep.2013.10.021

[240] Smith PF, Bullock JM, Booker BM, Haas CE, Berenson CS, Jusko WJ. Induction of imatinib metabolism by hypericum perforatum. Blood. 2004;104(4):1229–30. 15294859 10.1182/blood-2004-04-1240

[241] Wang Z, Sun X, Feng Y, Wang Y, Zhang L, Wang Y, et al. Dihydromyricetin reverses MRP2-induced multidrug resistance by preventing NF-kappaB-Nrf2 signaling in colorectal cancer cell. Phytomedicine. 2021;82: 153414. 33461143 10.1016/j.phymed.2020.153414

[242] Zhang XQ, Ding YW, Chen JJ, Xiao X, Zhang W, Zhou L, et al. Xiaoaiping injection enhances paclitaxel efficacy in ovarian cancer via pregnane X receptor and its downstream molecules. J Ethnopharmacol. 2020;261: 113067. 32505840 10.1016/j.jep.2020.113067

[243] Kapelemera AM, Uang YS, Wang LH, Wu TY, Lee FY, Tai L, et al. Pharmacokinetic herb-drug interactions of Xiang-Sha-Liu-Jun-Zi-Tang and paclitaxel in male sprague dawley rats and its influence on enzyme kinetics in human liver microsomes. Front Pharmacol. 2022;13: 858007. 35450043 10.3389/fphar.2022.858007 PMC9016196

